# Expression of a fungal ferulic acid esterase in alfalfa modifies cell wall digestibility

**DOI:** 10.1186/1754-6834-7-39

**Published:** 2014-03-20

**Authors:** Ajay Badhan, Long Jin, Yuxi Wang, Shuyou Han, Katarzyna Kowalczys, Daniel CW Brown, Carlos Juarez Ayala, Marysia Latoszek-Green, Brian Miki, Adrian Tsang, Tim McAllister

**Affiliations:** 1 Agriculture and Agri-Food Canada, Lethbridge Research Centre, 5403 1st Avenue South, Lethbridge, AB T1J 4B1, Canada; 2 Agriculture and Agri-Food Canada, Southern Crop Protection and Food Research Centre, London, ON N5V 4T3, Canada; 3 Canadian Centre for Agri-Food Research in Health and Medicine, St Boniface Hospital Research Centre, Winnipeg, MB R2H 2A6, Canada; 4 Biology Department, Concordia University, Montreal, QC H4B 1R6, Canada

**Keywords:** Cell wall, Ferulic acid esterase, Animal nutrition, Lignin, Digestibility, Transgenic alfalfa

## Abstract

**Background:**

Alfalfa (*Medicago sativa*) is an important forage crop in North America owing to its high biomass production, perennial nature and ability to fix nitrogen. Feruloyl esterase (EC 3.1.1.73) hydrolyzes ester linkages in plant cell walls and has the potential to further improve alfalfa as biomass for biofuel production.

**Results:**

In this study, *faeB* [GenBank:AJ309807] was synthesized at GenScript and sub-cloned into a novel pEACH vector containing different signaling peptides to target type B ferulic acid esterase (FAEB) proteins to the apoplast, chloroplast, endoplasmic reticulum and vacuole. Four constructs harboring *faeB* were transiently expressed in *Nicotiana* leaves, with FAEB accumulating at high levels in all target sites, except chloroplast. Stable transformed lines of alfalfa were subsequently obtained using *Agrobacterium tumefaciens* (LBA4404). Out of 136 transgenic plants regenerated, 18 independent lines exhibited FAEB activity. Subsequent *in vitro* digestibility and Fourier transformed infrared spectroscopy (FTIR) analysis of FAEB-expressing lines showed that they possessed modified cell wall morphology and composition with a reduction in ester linkages and elevated lignin content. Consequently, they were more recalcitrant to digestion by mixed ruminal microorganisms. Interestingly, delignification by alkaline peroxide treatment followed by exposure to a commercial cellulase mixture resulted in higher glucose release from transgenic lines as compared to the control line.

**Conclusion:**

Modifying cell wall crosslinking has the potential to lower recalcitrance of holocellulose, but also exhibited unintended consequences on alfalfa cell wall digestibility due to elevated lignin content. The combination of efficient delignification treatment (alkaline peroxide) and transgenic esterase activity complement each other towards efficient and effective digestion of transgenic lines.

## Background

Plant cell walls containing cellulose, hemicellulose, pectin and lignin are the most abundant organic resource on the planet [1]. Due to the limited availability of fossil carbon, as well as environmental concerns, comprehensive utilization of lignocellulosics for fuel and chemical production has currently raised much interest. Recalcitrance to scarification is a major limitation for conversion of lignocellulosic biomass to biofuels [2]. Plant cell walls represent a major source of nutritional energy for ruminants, but unfortunately with many types of forage, less than 50% of the cell wall fraction is readily digested and utilized by the ruminant host. Substantial benefits would be realized if a greater percentage of this potential energy was made available for fermentation through an increase in the digestibility of the cell wall fraction [3].

Plants have evolved effective mechanisms for resisting assault on their cell walls from the microbial and animal kingdoms. This intrinsic property underlies what has been termed ‘recalcitrance’, creating technical barriers to the cost effective transformation of lingo-cellulosic biomass into fermentable sugars. The natural factors believed to contribute to the recalcitrance of ligno-cellulosic feedstock to chemicals or enzymes include: 1) the epidermal tissue of the plant body, particularly the cuticle and epicuticular waxes; 2) the degree of lignification; 3) the structural heterogeneity and complexity of cell wall constituents such as microfibrils and matrix polymers; 4) the challenges for enzymes acting on an insoluble substrate; and 5) the inhibitors to subsequent fermentation that exist naturally in cell walls or are generated during the conversion process [4]. These chemical and structural features of biomass affect liquid penetration and/or enzyme accessibility and activity and, thus, conversion costs. These same constraints are equally relevant to the utilization of forage by ruminants.

Physical and chemical pretreatments have been developed to optimize the separation of lignin and cell wall polysaccharides from different feedstocks [5,6]. Plant fiber engineering is also seen as a route to improve feedstock characteristics and further reduce the energy and cost of refining biomass [7-9]. Several publications also describe the potential of modifying plant cell wall composition by altering the expression of endogenous enzymes involved in cell wall synthesis [10-12]. Similarly, cell wall composition, architecture and susceptibility to downstream processing can be improved through transgenic expression of exogenous carbohydrate-active enzymes (CAZymes) *in planta*. Expression of the type A ferulic acid esterase from *Aspergillus niger* (An*Fae*A) in the grasses *Lolium multiforum*[13] and *Festuca arundinacea*[14] has been explored as a means of reducing cell wall crosslinking and thus increasing ruminal digestibility. The vast majority of An*Fae*A transformed plant lines showed significant decreases in the cell wall content of ferulic acid and diferulate as compared to controls. Recently, Tsai *et al*. [15] reported altered intermolecular crosslinking within plant cell walls as a result of constitutive expression of a fungal glucuronoyl esterase in *Arabidopsis*. Likewise, improved rumen digestibility of forage could also increase their potential as raw materials for biofuel production. A recent study showed that simple modification of the cell wall in alfalfa dramatically increased its susceptibility to hydrolysis [16].

The aims of our study were to express the fungal (*A. niger*) feruloyl esterase B gene (*faeB*) in alfalfa by stable transformation, and to evaluate apoplast (A), chloroplast, endoplasmic reticulum (ER) and vacuole (V) targeted transgenic lines as a new feedstuff for ruminants as well as a biomass resource for cellulosic biofuel production. *faeB* was preferred over the feruloyl esterase A gene (*faeA*) considering its suitability for hydrolyzing ferulic acid esters in dicot cell walls (alfalfa), as dicots contain ferulated pectic polysaccharides as compared to grasses where ferulic acid is esterified to arabinoxylans. FAEB has been reported to be highly active against esterified pectin, while FAEA prefers esterified xylan.

## Results

### Transgenic plants

The number of plants regenerated from *in vitro* culture ranged from 33 (for the *faeB* targeted to endoplasmic reticulum) to 168 (for the *faeB* targeted to apoplast) using kanamycin resistance screening, with a total of 136 of these successfully rooted and established in the greenhouse. The efficiency of stable transformation, calculated as the percentage of explants (petioles) producing stable transgenic plants, varied with the construct. The recovery rate, based on PCR detection, of *faeB*-apoplast transgenic plants was found to be the highest at 54.2%, while the recovery rate of *faeB*-ER transgenic plants was the lowest at 8.3%. Transformation efficiencies of alfalfa for the *faeB* construct targeted to the chloroplast was 20.8% and 30.0% for *faeB* targeted to the vacuole (Additional file 1).

### *faeB* expression *in planta*

**Figure 1:**
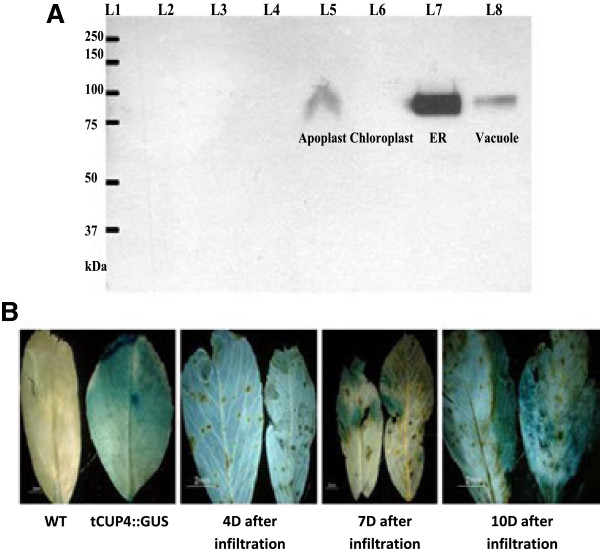
**Gene expression analysis. (A)** Western blot showing transient expression of *faeB* proteins in tobacco leaves. L1, protein ladder (catalogue number 161-0374; Bio-Rad, Hercules, CA, USA); L2, uninoculated leaf proteins; L3, proteins of tobacco leaves infected with LBA4404 (no construct); L4, proteins of tobacco leaves infected with agro harboring pEACH 5,103; L5, proteins of tobacco leaves infected with agro harboring *faeB*-apoplast; L6, proteins of tobacco leaves infected with agro harboring *faeB*-chloroplast; L7, proteins of tobacco leaves infected with agro harboring *faeB*-ER; and L8, proteins of tobacco leaves infected with agro harboring *faeB*-vacuole. **(B)** Transient expression of GUS in alfalfa leaves. tCUP:GUS was stably transformed into alfalfa plants (obtained from Dr Lining Tian, Agriculture and Agri-Food Canada London, ON, Canada), served as a positive control. ER, endoplasmic reticulum; GUS, β-glucuronidase.

As a reporter gene for evaluating and optimizing the protocol for transient expression of the introduced gene *in planta,* we monitored transient expression of β-glucuronidase (GUS) in alfalfa leaves. To confirm the functionality of the construct and its ability to express in dicotyledonous plants in the targeted organelle, we used Western blot analysis of transiently transformed tobacco lines (Figure 1A) prior to stable transformation, and showed that the codon-optimized *faeB* genes were expressed in the apoplast, endoplasmic reticulum and vacuole with expression being highest in the endoplasmic reticulum. The gene did not express in the chloroplast. Transient GUS expression could be seen 5 days after infiltration with expression levels plateauing 10 days after infiltration (Figure 1B).

**Figure 2:**
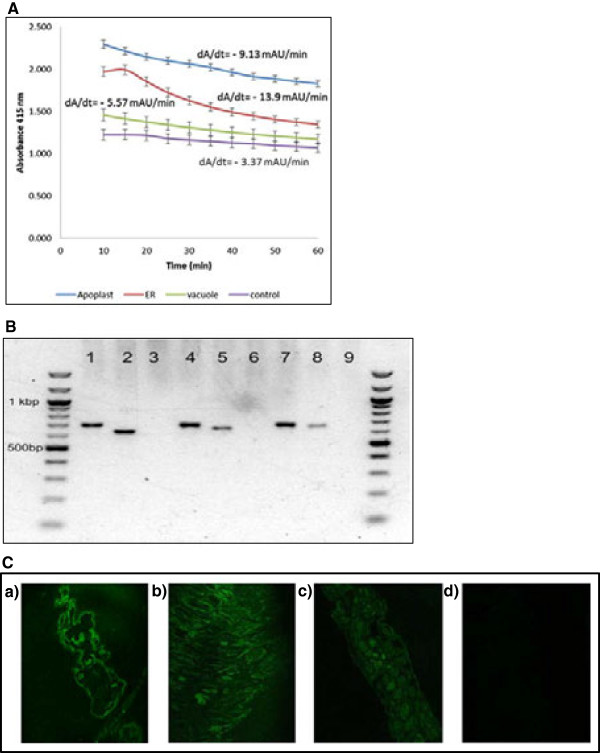
**FAEB activity assay, PCR validation of *faeB *and immunocytochemical localization of FAEB. (A)** FAEB activity as indicated by the change in absorbance over 60 min as a result of the hydrolysis of ethyl ferulate. Data represents three absorbance readings from the same extract from a single plant. Error bars indicate relative standard error. Apoplast (A), transgenic line 3A; control, wild type plant; endoplasmic reticulum (ER), transgenic line 28ER and vacuole (V), transgenic line 2 V. **(B)** PCR validation of *faeB* gene in transgenic lines. Apoplast-expressing sample: lane 1, *nptII* (apoplast); lane 2, *faeB*-apoplast and lane 3, wild type negative control for *fa*e*B*-apoplast. ER-expressing sample: lane 4, *nptII* (ER); lane 5, *faeB*-ER and lane 6, wild type negative control for *faeB*-ER. Vacuole-expressing sample: lane 7, *nptII* (vacuole); lane 8, *faeB*-vacuole and lane 9, wild type negative control for *faeB*-vacuole. **(C)** Immunocytochemical localization to confirm recombinant protein expression in: **(a)** apoplast; **(b)** endoplasmic reticulum; **(c)** vacuole and **(d)** non-transgenic control in alfalfa leaves. A, apoplast; ER, endoplasmic reticulum; FAEB, type B ferulic acid esterase; V, vacuole.

Of the 136 stably transformed lines based on PCR detection of the *faeB* gene and Southern blot (Additional file 2), 18 showed detectable levels of *faeB* activity (Figure 2A,B; data shown for representative lines 2 V, ER28 and 3A covering one line from each genotype) using a microplate rapid screening assay [17]. Of the 18 *faeB*-expressing lines, 11 were targeted to apoplast, five targeted the vacuole and two targeted the endoplasmic reticulum. Transformed plants were clonally multiplied by taking cuttings of young growing shoots and rooted in a moist sand bed. Figure 2B shows PCR confirmation of expression of the *faeB* transcript in transgenic lines used for further study. Representative transgenic lines were assessed using a whole-mount immunolocalization technique after Sauer *et al*. [18] to confirm recombinant protein localization in the target (endoplasmic reticulum, apoplast and vacuole) area and, as shown in Figure 2C, expression of genes was confirmed in the targeted plant cell compartment.

### *In vitro* digestion by mixed rumen microorganisms

**Figure 3:**
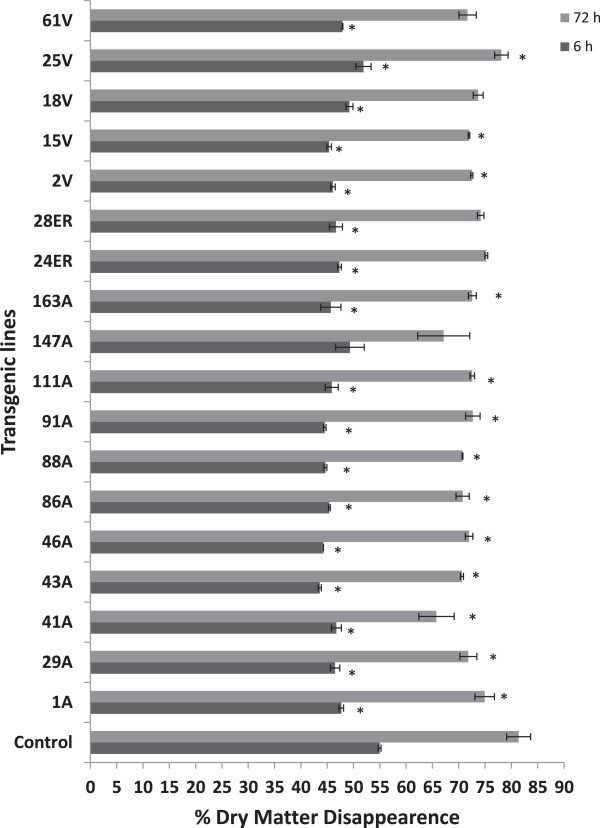
**Effect of FAEB activity on *in vitro *dry matter disappearance (IVDMD) of control and various transgenic lines incubated with mixed ruminal fluid.** Bars indicate standard error. *Differs to control at *P* < 0.05. Repeated two times with triplicate samples per incubation. A, apoplast; ER, endoplasmic reticulum; FAEB, type B ferulic acid esterase; IVDMD, *in vitro* dry matter disappearance; V, vacuole.

*In vitro* dry matter disappearance (IVDMD, %) was lower (*P* < 0.05) for most of the transgenic lines as compared to the non-transformed clonal line after 6 h and 72 h of incubation with mixed ruminal fluid (Figure 3). This result suggests a possible negative effect of transgenic esterase activity on digestibility of the plant cell wall, an unanticipated outcome. Total volatile fatty acid (VFA) concentrations arising from digestion (Additional file 3) were observed to be similar for the parental and transgenic lines. *In vitro* ammonia production was lower in transgenic as compared to non-transgenic lines (Additional file 4), suggesting that the transformation process also altered the fermentability of alfalfa protein.

### Cell wall analysis using Fourier transformed infrared spectroscopy (FTIR)

**Figure 4:**
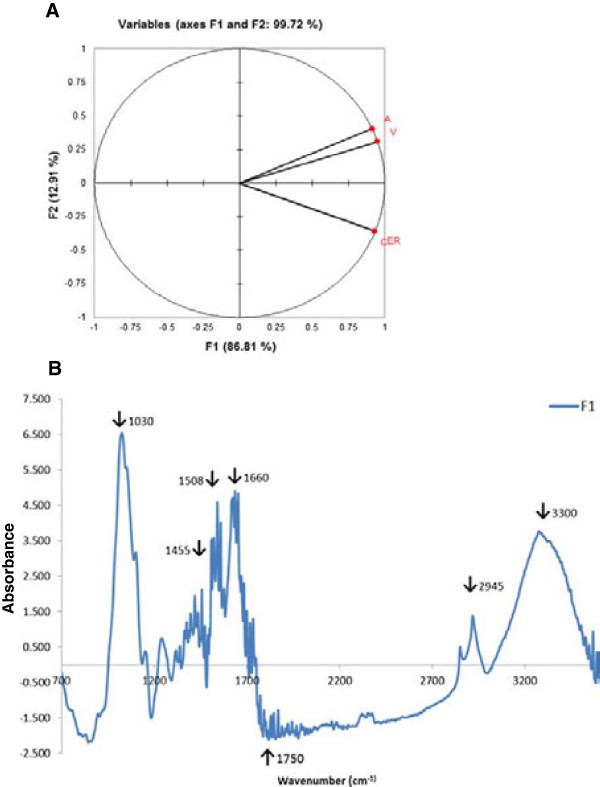
**Fourier transformed infrared spectroscopy (FTIR) data analysis. (A)** PCA of FTIR data. **(B)** Loading of factor score (F1) corresponding to PCA major spectral differences between transgenic and wild type lines. Apoplast (A), average spectrum of 43A, 41A and 1A; endoplasmic reticulum (ER), average spectrum of 24ER and 28ER; and vacuole (V), average spectrum of 61 V, 15 V and 2 V. C, control.

To gain better insight into alterations in cell wall architecture, we further analyzed the cell walls of transgenic and non-transgenic alfalfa using Fourier transformed infrared spectroscopy (FTIR). FTIR spectra from cell walls of transgenic lines (spot (A) apoplast: average of spectrum from transgenic line 43A, 41A and 1A; spot (ER) endoplasmic reticulum: average of spectrum from transgenic line 28ER and 24ER; spot (V) vacuole: average of spectrum from transgenic line 61 V, 15 V and 2 V) and the control (C) line were used for data analysis. Principal component analysis (PCA) of spectra (Figure 4A), showed that vacuole and apoplast transgenic lines grouped separately from endoplasmic reticulum and control lines, suggesting compositional differences among lines. However, although the correlation map (Figure 4A) projected endoplasmic reticulum as being closely related to the control, the correlation matrix value of 0.9 for the control versus endoplasmic reticulum is indicative of significant compositional differences between these lines, a result confirmed by the correlation map of axis F1 versus F3 (Additional file 5). Loading factor score (F1) corresponding to PCA (Figure 4B) showed a characteristic peak of an ester bond at 1,750, 1,735 and 1,715 cm^-1^ indicating differences in the degree of esterification between the control and transgenic lines [19,20]. Furthermore, bands specific for lignin (1,030, 1,508, 1,660 and 2,945 cm^-1^) also differed between the transgenic and control lines [19]. A broad peak between 3,200 to 3,400, 2,915 and 1,462 cm^-1^ was also indicative of elevated wax content in the transgenic as compared to the parental cell wall (Figure 4B) [20]. Elevated lignin content of transgenic lines was also evident from the peaks at 1,462 cm^-1^ (representing substituted H and HOC bending of aromatic skeletal vibration), 1,508 cm^-1^ (Aryl ring structure), 1,608 cm^-1^ (indicating existence of aromatic skeletal vibration, guaiacyl-syringyl type), 1,620 cm^-1^ (ring conjugated C = C structure of coniferaldehyde) and 1,660 cm^-1^ (ring conjugated C = C structure of coniferyl alcohol; C = O structure of coniferaldehyde) (Figure 4B) [19,20].

To investigate the effect of these variations in cell wall composition on rumen digestibility, we further analyzed the residue remaining after 72 h of *in vitro* rumen digestion by mixed rumen microorganisms. Digital subtraction of FTIR spectrum of residual cell walls (control-transgenic lines) after 72 h of *in vitro* digestion showed that the residue from transgenic lines with esterase activity targeted to the endoplasmic reticulum exhibited significantly higher levels of hemicellulose (1,100 to 1,300 cm^-1^) as compared to the control (Additional file 6A), whereas residue remaining when esterase was targeted to the apoplast had higher residual cellulose and hemicellulose (900 to 1,050 cm^-1^) content as compared to the control (Additional file 6B). To follow the progression of digestion we further subtracted FTIR spectra of residual cell walls after 72 h of incubation from FTIR spectra of residue of cell walls after 6 h of incubation. Results indicated that the majority of the non-transgenic cell walls were digested between 6 h and 72 h (Additional file 7A). In contrast, the transgenic lines were observed to contain much higher levels of undigested cellulose and hemicellulose in the residue (Additional file 7B,C).

### Acetyl bromide solubilized lignin content, total sugar content, total uronic acid and cell wall phenolic analysis

**Figure 5:**
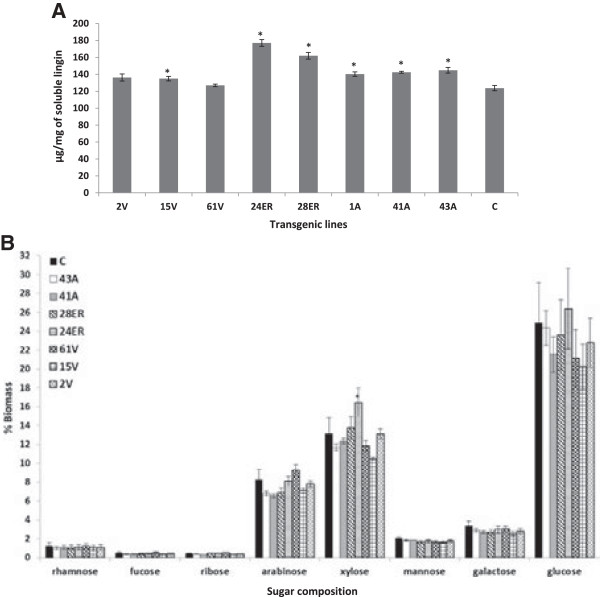
**Lignin content and sugar compositional analysis. (A)** Acetyl bromide soluble lignin content. Bars indicate standard error of mean (n = 3). *Differs to control at *P* 0.05. **(B)** Sugar composition of control and transgenic alfalfa. Sugars were quantified as alditol acetate derivatives by GC-MS. Error bars indicate standard deviation (n = 3) of technical replicates from ground samples pooled from 50 to 60 whole plants per genotype. Alfalfa lines indicate expression of *faeB* in endoplasmic reticulum (ER), 24ER and 28ER; apoplast (A), 43A, 41A and 1A; and vacuole (V), 61 V, 15 V and 2 V. A, apoplast; C, wild type control; ER, endoplasmic reticulum; GC-MS, gas chromatography–mass spectrometry; V, vacuole.

**Figure 6:**
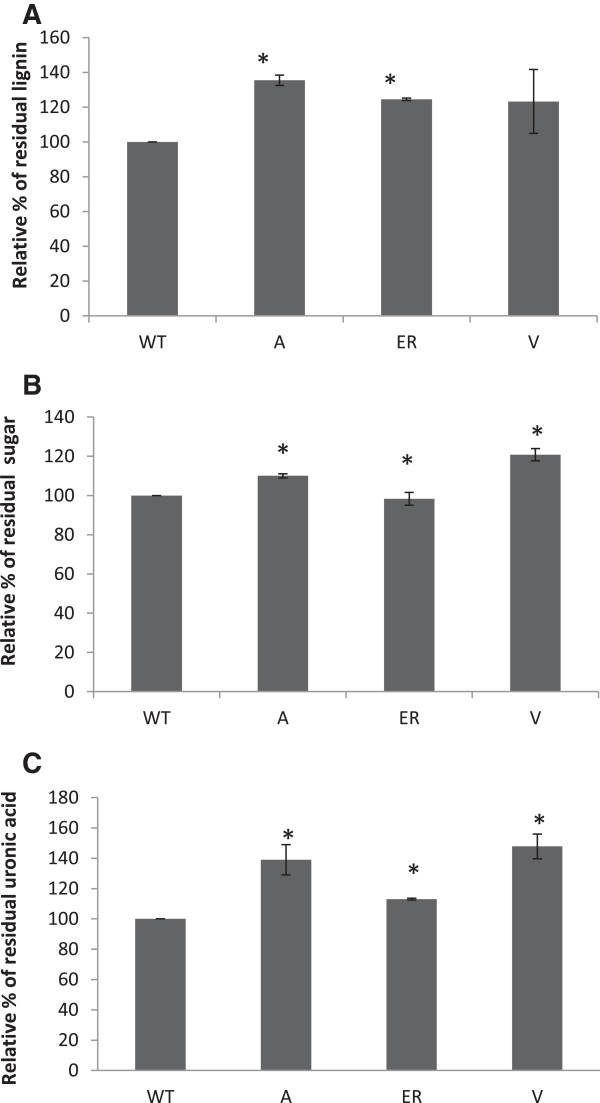
**Residual lignin, total sugar and uronic acid content of cell walls after 72 h of *in vitro *digestion.** .Relative percentage of **(A)** residual lignin, **(B)** sugar and **(C)** uronic acid, which equal the percentage of lignin, sugar and uronic acid, respectively, remaining after 72 h of digestion relative to that of the control (control being 100%). Bars indicate standard error of mean (n = 3). *Differs to control at *P* 0.05. Apoplast (A), *fa*e*B*-apoplast (average of 43A, 41A and 1A); endoplasmic reticulum (ER), *fa*e*B*-ER (average of 24ER and 28ER); and vacuole (V), *fa*e*B*-vacuole (average of 61 V, 15 V and 2 V). A, apoplast; ER, endoplasmic reticulum; V, vacuole; WT, wild type.

**Figure 7:**
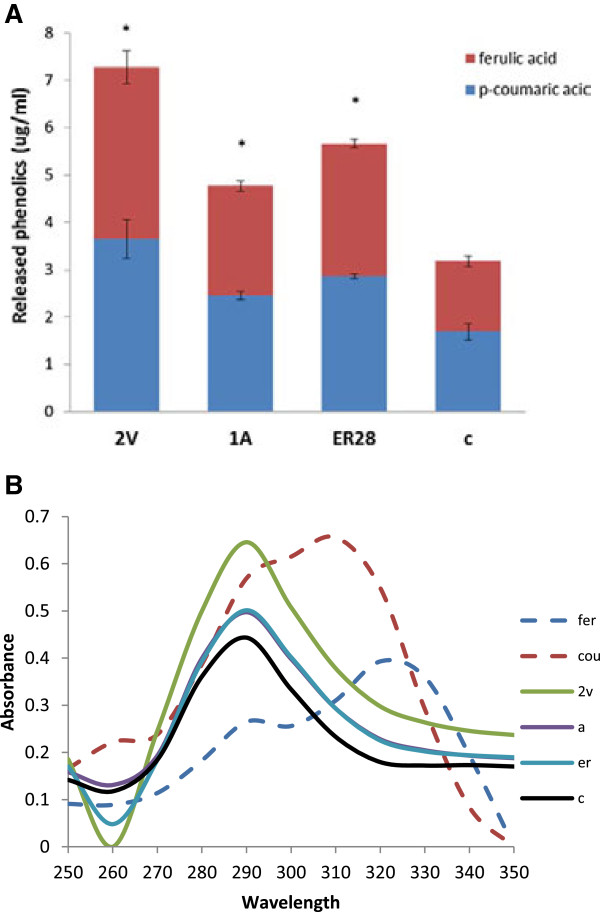
**Cell wall extractable phenolics analysis. (A)** Release of ferulic acid and *p*-coumaric acid as a result of extraction with 1 M NaOH. **(B)** UV spectra of lignin fraction extracted with 1 M NaOH from control and three representative transgenic lines from endoplasmic reticulum (ER), apoplast (A), vacuole (2 V). and C, control.

Most transgenic lines (43A, 41A, 1A, 28ER, 24ER and 15 V) exhibited higher (*P* < 0.05) levels of acetyl bromide soluble lignin as compared to non-transgenic lines (Figure 5A). Moreover, relative lignin content of residual cell walls after 72 h of digestion was also observed to be higher for transgenic lines (Figure 6A). Although the total sugar composition of sulfuric acid extracted cell wall fractions were observed to be similar among forage lines (Figure 5B and Additional file 8), the total sugar and uronic acid content were higher in residual cell walls of transgenic lines after 72 h of *in vitro* digestion as compared to the controls (Figures 6B,C). Transgenic lines with targeted esterase expression in the vacuole showed the highest release of phenolics (Figure 7A). Spectra showed characteristic peaks for ferulic and *p*-coumaric acid at 290 and 330 nm and 290 and 310 nm, respectively. Elevated ferulic and *p*-coumaric acid in transgenic lines were evident from higher peaks at 290, 310 and 330 nm as compared to the control line (Figure 7B).

### Enzyme hydrolyzability assay

**Figure 8:**
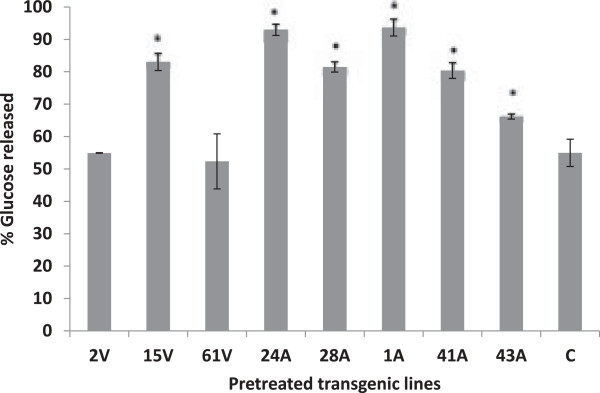
**Effect of delignification by alkaline peroxide pretreatment on glucose released from control and transgenic lines as a result of hydrolysis with commercial enzyme preparations (Accellerase 1500).** Bars indicate standard error of mean (n = 8). *Differs to control at *P* <0.05. Alfalfa lines indicate expression of *faeB* in endoplasmic reticulum (ER), 24ER and 28ER; apoplast (A), 43A, 41A and 1A; and vacuole (V), 61 V, 15 V and 2 V. A, apoplast; C, wild type control; ER, endoplasmic reticulum; V, vacuole.

As *in vitro* rumen digestion suggested a greater recalcitrance of transgenic lines it was proposed that cell wall carbohydrates in these lines could be made more accessible by delignification using alkaline hydrogen peroxide (AHP). To further explore this possibility, the AHP pretreated control and transgenic lines (43A, 41A, 1A, 28ER, 24ER, 61 V, 15 V and 2 V) were digested with a commercial mixed enzyme preparation (Accellerase 1500 containing endoglucanase: 2,200 to 2,800 carboxymethylcellulose (CMC) U/g and beta-glucosidase: 450 to 775 *p*-nitrophenyl-β-D-glucopyranoside (pNPG) U/g). In agreement with incubations with mixed rumen microbes, glucose release from untreated control lines did not differ from untreated transgenic lines. In contrast, after pretreatment most transgenic lines released more glucose as compared to the control (Figure 8). Compared to the control, pretreated transgenic line 1A yielded 39% more glucose, whereas transgenic lines 24 ER and 15 V released 38% and 29% more glucose, respectively.

## Discussion

Alfalfa is a valuable forage crop, primarily because of its high protein content and palatability for ruminants. However, the fiber fraction of alfalfa is often poorly digested and in fact can be even more lignified than grasses [21]. Acetyl group and linkages of uronic acid side chains to phenolic material have been identified as factors that limit the ruminal digestion of alfalfa [22]. Similarly, Giraldo *et al*. [23], Nsereko *et al*. [24] and Wang *et al*. [25] reported ester linkages between hemicellulose and lignin as the rate limiting factor in the ruminal digestion of plant cell walls. Hydrolyzing these linkages by chemical treatment with alkali is known to increase biodegradability and the nutritional value of low-quality feed. Reducing the level of crosslinking of cell wall carbohydrates is therefore a predictable way of improving forage quality through increases in the rate and possibly the extent of digestion [13].

In this study we developed alfalfa plants with transgenic ferulic acid esterase activity targeted to the apoplast, endoplasmic reticulum and vacuole. The targeting to these specific cellular organelles was based on previous work using tobacco [26], which showed expression of recombinant proteins in these organs at a detectable level, particularly in the endoplasmic reticulum. The cell wall composition of transgenic lines assessed in this study (43A, 41A, 1A, 28ER, 24ER, 61 V, 15 V and 2 V) was altered as a result of transgenic esterase activity to varying magnitudes. Interestingly, it seems that transgenic expression of ferulic acid esterase in alfalfa reduced its *in vitro* ruminal digestibility (Figure 3). This is likely attributed to the increase in lignin content of transgenic lines of alfalfa as indicated by FTIR spectrum analysis and wet chemistry. Likewise, the major monolignol constituents of lignin, that is the hydroxycinnamic acids, ferulic and *p*-coumaric acid, were found to be elevated in transgenic lines as compared to the control, likely a reflection of their higher lignin content. The increase in lignin content of transgenic lines observed in this study is in agreement with a recent report of elevated lignin levels in another dicot, *Arabidopsis*, expressing a fungal glucuronoyl esterase [15]. These results suggest that targeted expression of *faeB* in the endoplasmic reticulum/golgi apparatus (where ferulated arabinoxylan and pectin are formed prior to secretion to the cell wall) increased free ferulate content and the synthesis of lignin in transgenic lines. This hypothesis is supported by the finding that phenolic polymers are produced when levels of phenylpropanoid pathway intermediates or end products are increased [27]. Elevated wax content may also have contributed to the higher recalcitrance of transgenic lines. The visible phenotypic characteristics of recovered lines did not seem to be affected due to this change in lignin or wax content as transgenic plants were largely indistinguishable from controls, although expression of the introduced gene did impact the recovery of plants from cell culture. For dicots, strong negative correlations have been shown between initial lignin content and cellulose digestibility in alfalfa pretreated with dilute acid [16]. Phenolic components of the plant cell wall, especially *p*-coumaric acid, ferulic acid and *p*-hydroxybenzaldehyde have been reported to inhibit the growth of rumen microorganisms and cellulase activity [28,29]. Furthermore, it has been reported that lignin physically hinders the accessibility of enzymes to cellulose [30]. Moreover, soluble lignin-derived compounds may also cause enzyme inhibition [31]. The tendency of cellulase to bind irreversibly to lignaceous surfaces restricts conformational shifts in enzymes, which is crucial for the degradation of insoluble polysaccharides [32].

Notably then, delignification pretreatments such as AHP resulted in higher glucose release from transgenic as compared to the control line, a reflection of low recalcitrant core hemicellulose and cellulose constituents in transgenic lines. Previously, it has been reported that AHP not only effectively delignified cell walls but also improved overall hydrophilicity of the cell wall matrix, thereby increasing water and enzyme penetration [33]. Likewise, the relative improved digestibility of transgenic lines observed in this study after AHP treatment can be attributed to a combination of efficient delignification by AHP and transgenic esterase activity, leading to improved accessibility of cellulase and/or xylanase to low recalcitrant core cell wall components. The approach adopted here therefore indicates the potential utility of modified cell wall crosslinking for improved and effective digestibility of plant cell walls for ethanol production and use by ruminants.

## Conclusions

The results suggested reduced esterification, but increased lignin content in transgenic lines which possibly correlated to decreased rate of digestibility by ruminal microorganisms. Interestingly, alkaline peroxide pretreatment followed by commercial enzyme hydrolysis resulted in higher glucose release from transgenic lines than from wild type plants suggesting higher digestibility of the holocellulose content for transgenic lines after lignin removal. The present study demonstrates that expression of some genes involved in cell wall digestion can have unintended consequences on the cell wall digestibility of alfalfa.

## Methods

### *faeB* gene

Feruloyl esterase B (*faeB*) from *A. niger* is composed of 521 amino acids. Among them, 18 amino acids in the N-terminus function as a signal peptide that aides in the secretion of *faeB*. To target the expression of *faeB* to specific plant organelles, we cleaved the N-terminal signal peptide coding region, and replaced it with PR1b to target the proteins to the endoplasmic reticulum [34] and Rubisco leading the peptide to target expression to the chloroplast [26]. Endoplasmic reticulum retention signal KEDL [35] and vacuole retention signal CTPP (from R Menassa, Agriculture and Agri-Food Canada, London, ON, Canada) were fused immediately after the protein to target the protein to the endoplasmic reticulum and vacuole, respectively. The c-Myc tag was used for protein purification and StrepII was used for detection. The schematic maps of the four *faeB* constructs are shown in Additional file 9. The codon usage of the *faeB* gene was optimized based on alfalfa codon usage preference with *faeB* synthesized at GenScript (Township, NJ, USA). The sequences of synthetic *faeB* genes are shown in Additional file 10.

### Plasmid construction

The transformation vector pPZP100 [36] was obtained from P Maliga (Rutgers University, New Brunswick, NJ, USA), and modified. In the pPZP100 vector, the CmR selectable marker gene was replaced with NPTII for selection of bacteria on kanamycin. The CmR gene generated instability in the vector, whereas it was stable with kanamycin (data not shown). In pEACH the non-mutated form of the NPTII gene [37] was inserted into the CmR gene, leaving the interrupted CmR gene sequence in the vector (Additional files 11 and 12). The MYB recognition sequence [38] allowed the excision of all sequences cloned into the multicloning site and acted as filler DNA to provide distance from the transfer DNA (T-DNA) borders [39], thereby minimizing interactions with elements at the insertion sites.

The plant selectable marker gene for kanamycin resistance was regulated by the enhanced tCUP4 promoter and ARBC terminator with the terminator adjacent to the left T-DNA border. Any deletions occurring at the left border would be selected against during plant tissue culture in the presence of kanamycin.

Genes regulated by the enhanced tCUP4 promoter and the PIN terminator were inserted in the same orientation as the selectable marker gene. The position of the enhanced tCUP4 promoter at the right border reduced the likelihood of promoter interactions with sequences within the adjacent insertion site. Enhanced tCUP4 has been shown to have no effect on the expression of adjacent genes, whereas the commonly used 35S promoter and super-promoter have been shown to interact with elements at the insertion site over large distances [40].

The *Eco*R I-*Hind* III fragment from the pEACH vector was released from pEACH 5,103 and subcloned into pUC18. In the resulting pUC18 vector, the synthetic *faeB* genes with protein signaling peptides were inserted between *Bam*H I and *Xba* I sites. Finally, the *Eco*R I-*Hind* III fragment from pUC18 vector was cloned back into pEACH 5,103, resulting in *faeB*-apoplast, *faeB*-chloroplast, *faeB*-ER and *faeB*-vacuole constructs, respectively. All inserts were sequenced to confirm identity to original sequences.

The schematic map of the four *faeB* constructs is shown in Additional file 9.

### *faeB* transient expression in tobacco

The four *faeB* expression constructs were integrated into *Agrobacterium tumefaciens* strain LBA4401 using electroporation and were transiently expressed in 5- to 6-week-old *Nicotiana benthamiana* as described by Joensuu *et al*. [41]. Total proteins were extracted from tobacco leaves infected with LBA4404 *Agrobacterium*, LBA4404 transformed with pEACH 5,103 (GUS-intron), *faeB*-apoplast, *faeB*-chloroplast, *faeB*-ER and *faeB*-vacuole vectors, respectively, and 25 μg of each protein was run on SDS-PAGE gel. Expressed *faeB* proteins were detected using the primary antibody anti-c-Myc (GenScript; 1,500 ×) and the secondary antibody, goat anti-mouse immunoglobulin G (IgG) with horseradish peroxidase (Bio-Rad; 3,000 ×).

### Tissue culture and plant transformation

Donor plants of alfalfa (*Medicago sativa* L.) genotype N.4.4.2 were propagated *in vitro* by subculturing individual nodes in 10 cm magenta containers containing 0.5 × Murashige and Skoog medium (MSO) [42]. The standard conditions for maintaining the cultures in a growth chamber were 25°C (day/night) with a photoperiod of 16 h at approximately 3,500 lux.

For alfalfa tissue culture and transformation, the procedures were performed as outlined in Han *et al*. [43]. Briefly, the petioles were cut to 1 cm lengths and pre-cultured on SH2K medium [44] for 48 h at 25°C with a photoperiod of 16 h. After pre-culture, explants were immersed (shortly for 2 to 5 seconds) in a suspension of *Agrobacterium* cells cultured overnight (OD_600_ = 0.6 to 0.8). After immersion, petioles were blotted onto filter paper and placed on SH2K medium and co-cultivated for 5 days in the dark. The petioles were then transferred to the medium used for co-cultivation containing 300 mg/l timentin, and incubated for 2 weeks. When callus formation was observed, calli were transferred onto SH2K medium containing 50 mg/l kanamycin and 300 mg/l timentin. Calli surviving 1 week on this selection medium were moved to medium containing 75 mg/l kanamycin and 300 mg/l timentin, and incubated for another 2 weeks. The calli were then transferred to the embryo induction medium BOi2Y [45,46] containing 300 mg/l timentin and 75 mg/l kanamycin, and incubated for 3 weeks in the light with a photoperiod of 16:8. Green elongated mature embryos with well-formed cotyledons were collected and transferred to 0.5 × MSO medium with 300 mg/l timentin and 75 mg/l kanamycin for 2 to 3 weeks. Germinated embryos were transferred to MSO containing 300 mg/l timentin and 25 mg/l kanamycin in magenta boxes. Well-established plants were transferred to soil. As a negative control, non-transformed explants were placed in SH2K medium with kanamycin (75 mg/l) to ensure effective selection of transformants (Additional file 13).

### Plant material

The control (non-transformed) and regenerated transgenic lines were multiplied in a greenhouse using cuttings of actively growing young shoots rooted in a moist sand bed, transferred to a soilless potting mixture (Pro-Mix, Premier Tech Horticulture, Rivière-du-Loup, QC, Canada) and grown in 200 cm plastic pots under seasonal greenhouse conditions, with daily watering and weekly fertilization (20 N:20P:20 K). Actively growing shoots were collected from all lines at the pre-bud vegetative stage, freeze-dried and stored at -20°C.

### Validation by PCR and southern hybridization

Genomic DNA was extracted from leaves of putative transformed and non-transformed alfalfa plants using a commercially available kit (REDExtract-N-Amp™ Plant PCR Kit; Sigma-Aldrich, St Louis, MO, USA). The integration of transgenes into the alfalfa genome was confirmed by PCR with primers targeting the *nptII* and *faeB* genes. Additionally, a common tCUP4 promoter forward primer was used in combination with transgene specific reverse primers for *faeB-*apoplast, *faeB-*chloroplast, *faeB-*ER and *faeB-*vacuole. For amplification of the *nptII* gene, a 699 bp fragment was amplified using the forward primer (5’GAGGCTATTCGGCTATGACTG3’) and the reverse primer (5’ATCGGGAGCGGCGATACCGTA3’). The primers for amplification of *faeB* gene fragments of four constructs were as follows. For the *fae*B-apoplast construct, the forward primer was (5’ACGGTGGAGAGGCTGATA3’) and the reverse primer was (5’GGATGACTCCAAAGATCCTC3’), generating a product of 652 bp. For the *fae*B-chloroplast construct, the forward primer was (5’CTGCTGCTGTTGCAACAAGG3’) and the reverse primer was (5’GGAAAGCACCCCATGA3’), generating a product of 765 bp. For the *fae*B-vacuole construct, the forward primer was (5’ACGGTGGAGAGGCTGATA3’) and the reverse primer was (5’CCTTACATAGTAACAAGCAAACCG3’), generating a product of 695 bp. For the *faeB*-ER construct, the forward primer was (5’ACGGTGGAGGCTGATA3’) and the reverse primer was (5’GGATCCTTAAAGTTCATCTT3’), with PCR product size of 680 bp. The conditions of PCR were set as follows: 94°C, 5 min; 30 cycles at 94°C, 30 sec; 58°C, 30 sec; 72°C, 30 sec; and a final extension at 72°C, 5 min. The primers used for amplification of the tCUP4 promoter and transgene specific reverse primers for *faeB-*apoplast, *faeB-*chloroplast, *faeB-*ER and *faeB-*vacuole were used. The forward primer flanking tCUP4 promoter was (5’CGGCAGAATTTCCCTATATATATTTTTAATTCCCAAA3’) and the transgene specific reverse primers were as follows: *faeB-*apoplast reverse primer was (5’GGATGACTCCAAAGATCCTC3’), the size of PCR product was 1,809 bp; *faeB*- chloroplast reverse primer was (5’GGAAAGCACCCCATGA3’), generating a PCR product of 956 bp; *faeB*-ER reverse primer was (5’GGATCCTTAAAGTTCATCTT3’), the size of PCR product was 1,842 bp; and *faeB*-vacuole reverse primer was (5’CCTTACATAGTAACAAGCAAACCG3’), generating a PCR product of 1,695 bp. PCR conditions were as follows: 94°C, 5 min; 35 cycles at 94°C, 30 sec; 55°C, 30 sec; 72°C, 1 min 45 sec; and the final extension at 72°C, 5 min.

For Southern analysis, total genomic DNA was isolated from selected transgenic and control plants, then digested overnight with HindIII (New England Biolabs, Ipswich, MA, USA), with 10 μg of digested DNA being separated by agarose electrophoresis. DNA was transferred onto a Hybond-N membrane (GE Healthcare Life Sciences, Mississauga, ON, Canada) by capillary blotting. DNA was fixed to the membrane by UV crosslinking and probed with digoxigenin-labeled *faeB* prepared by PCR of plasmid DNA.

### *In situ* immunolocalization

*In situ* immunolocalization of transgenic protein in the different organelles was performed on young alfalfa leaf tissue from transgenic and control plants, according to the protocol described by Sauer *et al*. [18]. A 1:1,000 dilution of the 0.5 mg/ml stock of c-Myc anti-mouse antibody was used as the primary antibody and a 1:30 dilution of donkey anti-mouse IgG antibody stock (Molecular Probes, catalogue number A21202) labeled with Alexa Fluor 488 (Life Technologies, Carlsbad, CA, USA) was used as the secondary antibody.

### Feruloyl esterase activity screening

Enzyme was extracted by grinding 3 g of stem and leaf tissue in liquid nitrogen, followed by suspension in 10 ml of extraction buffer (0.1% PBS Tween-20, 2% PVPP, 1 mM EDTA, 1 mM PMSF, 1 μg leupeptin, 100 mM ascorbic acid, pH 7.4). Crude protein extract was recovered by centrifugation and concentrated using centrifugal filters (Centriprep YM-10; EMD Millipore, Billerica, MA, USA). The concentrate was resuspended in 3 ml of 2.5 mM 3-(N-morpholino)propanesulfonic acid (MOPS) buffer (pH 7.2) and reisolated by centrifugation into a final volume of less than 1 ml. For preliminary assessment of feruloyl esterase activity in transgenic lines, enzyme was qualitatively estimated using a microplate screening assay [17] by measuring the hydrolysis of ethyl ferulate (50 mM ethyl ferulate, 5 mM of *p*-nitrophenol in isopropanol, diluted in nine volumes of 2.5 mM MOPS, pH 7.2). To test the samples, 10 mg (in triplicate) of concentrated plant protein extracts in 20 μl of 2.5 mM MOPS (pH 7.2) were placed in each microplate well, and 100 μl of substrate was added immediately before readings. Decrease in absorbance at 415 nm at 30°C was recorded (from triplicates) every 5 min for 1 h. Relative enzyme activity was determined by linear regression of the decrease in absorbance versus time and slope of the regression equation (Figure 2A).

### *In vitro* ruminal incubation

*In vitro* ruminal incubations were performed in 125 ml serum vials fitted with rubber stoppers. Whole alfalfa plants were ground to pass a 1.0 mm screen, and weighed into filter bags (F57; Ankom, Macedon, NY, USA; 0.5 g per bag) and loaded into vials prior to addition of ruminal inoculum. Six cows with permanent rumen cannula, fed an alfalfa hay diet were used as rumen fluid donors. Cattle used in this study were cared for in accordance with standards of the Canadian Council on Animal Care (CCAC, 1993). Rumen fluid was collected 2 h after feeding from five different locations in the rumen-reticulum and strained through four layers of cheese cloth. Equal amounts of rumen fluid from each cow were combined, mixed with a mineral buffer [47] in a ratio of 1:2 to serve as inoculum. Incubations were initiated by dispensing 20 ml of inoculum under a stream of CO_2_ into vials containing each substrate in F57 filter bags. The vials were immediately sealed and affixed to a rotary shaking platform (125 rpm) in a 39°C incubator (model 39419-1; Forma Scientific, Marietta, OH, USA). Triplicate vials for each sample and blank were retrieved from the incubator after 6 h and 72 h of incubation and processed for determination of VFAs, ammonia and IVDMD as previously described [48].

Residue remaining in the filter bag was rinsed thrice with phosphate buffer (pH 7.0), dried at 55°C and weighed to estimate the IVDMD. Two subsamples of the liquid culture were taken (1.5 ml each) from each vial immediately after retrieval of the filter bags. One sample was transferred to a 2 ml microcentrifuge tube containing 126 μl of trichloroacetic acid (TCA; 65% w/v) and centrifuged at 14,000 × *g* for 10 min to precipitate particulate. The supernatant was transferred into 2.0 ml microcentrifuge tubes and stored at -20°C until analysis for ammonia by the phenyl-hypochlorite method [49]. Another sample was mixed with 0.3 ml of metaphosphoric acid (25% w/v), centrifuged at 14,000 × g for 10 min and the supernatant was analyzed for VFA as described by Wang *et al*. [50].

### Attenuated total reflectance Fourier transform infrared spectroscopy (ATR-FTIR)

Ground samples and residues from *in vitro* digestion of transgenic (43A, 41A, 1A, 28ER, 24ER, 61 V, 15 V and 2 V) and control plants were subjected to FTIR spectroscopy using ALPHA FT-IR spectrometer (Bruker Optics, Ettlingen, Germany) equipped with a platinum diamond attenuated total reflectance (ATR) attachment. Spectra were collected over 4,000 to 600 cm^-1^ with resolution of 4 cm^-1^ and 32 repetitious scans were averaged for each spectrum. The sample contact area was circular with a diameter of approximately 1.5 mm as the samples were pressed against the diamond crystal of the ATR device. Spectra were baseline corrected and area normalized manually using Opus software (Opus Software Limited, Grantham, UK). Averages of 43A, 41A and 1A spectra were used to represent apoplast lines, while representative spectra for endoplasmic reticulum and vacuole targeted lines were computed by averaging data from lines 28ER and 24ER and 61 V, 15 V and 2 V, respectively. Spectra were subject to PCA and digital subtraction data analysis (XLSTAT 2013.4 statistical software; Addinsoft, New York, NY, USA).

### Cell wall analysis

For each line, 50 to 60 whole plants were pooled and ground as described above. Ground samples (60 to 70 mg) were used to prepare alcohol insoluble residue (AIR) as described previously [33]. Briefly, ground material was sequentially extracted over a sintered glass funnel under vacuum with two volumes of 100 ml of ice cold 80% ethanol, 100% ethanol, chloroform:methanol (1:1) and 100% acetone. Starch was removed by treatment with Type II-A *Bacillus* α-amylase (Sigma-Aldrich; approximately 1,000 units/100 mg cell wall AIR) in 50 mM sodium phosphate buffer (pH 7.0) at 25°C in a shaking incubator for 48 h. De-starched samples were centrifuged (3,660 × *g* for 10 min at 25°C) and the pellet was subsequently washed thrice with deionized water and recovered by centrifugation (3,660 × *g* for 10 min at 25°C). The resulting pellets were suspended in 500 μl of acetone and evaporated with a stream of air at 36°C until dry. For total sugar analysis, triplicate AIR samples (5 mg) of each line (43A, 41A, 1A, 28ER, 24ER, 61 V, 15 V, 2 V and C) were hydrolyzed with 72% H_2_SO_4_ and the released sugars were quantitated by a combination gas chromatography - mass spectroscopy (GC-MS) of alditol acetate derivatives. The remains after trifluoroacetic acid (TFA) treatment were hydrolyzed in Updegraff reagent (acetic acid:nitric acid:water, 8:1:2 v/v) and used in an anthrone assay to quantify crystalline cellulose [51]. Uronic acid content in triplicate AIR samples of each representative line (43A, 41A, 1A, 28ER, 24ER, 61 V, 15 V, 2 V and C) were quantified by adapting the micro-assay of van den Hoogen *et al*. [52] and using galacturonic acid as a standard. An average of 43A, 41A and 1A, 28ER and 24ER and 61 V, 15 V and 2 V were used to represent apoplast lines (A), endoplasmic reticulum (ER) and vacuole (V) targeted lines, respectively.

To determine lignin content [53], approximately 1 mg of AIR samples (three replicates for each line, that is, 43A, 41A, 1A, 28ER, 24ER, 61 V, 15 V, 2 V and C) were solubilized in freshly prepared acetyl bromide solution (100 μl of 25% (v/v) acetyl bromide in glacial acetic acid) for 3 h at 50°C, with 2 M sodium hydroxide (400 μl) and 0.5 M hydroxylamine hydrochloride (70 μl) being added to stop the reaction. Absorbance at 280 nm was measured using Synergy HT multi-detection microplate reader (Biotek Instruments, Inc., Winooski, VT, USA). Cell wall phenolics were extracted according to Buanafina *et al*. [14] with minor modifications. Briefly, following the extraction of chlorophyll pigments with aqueous methanol, ester bound compounds were extracted from ground plant material (50 mg, three repeats) with 1 M NaOH (5 ml) followed by incubation at 25°C for 24 h in the dark. Aliquots of the mixture were combined with a 1.2 volume of 100 mM HCl and centrifuged (1,000 × *g*, 20 min). The supernatant was diluted with four volumes of methanol and UV spectrum was recorded between 200 to 400 nm.

### Enzyme saccharification assay

Alkaline peroxide pretreatment was performed as described by Banerjee *et al*. [54]. Briefly, 50 ml of 1% H_2_O_2_ was adjusted to pH 11.5 with 5 M NaOH and mixed with 1 g of ground AIR plant material (as prepared above) in a 250 ml Erlenmeyer flask. Final concentrations were 1% H_2_O_2_ (300 mM), 0.8% NaOH (200 mM) and 2% biomass. The flasks were incubated at 24°C with shaking at 90 rpm for 24 h. The slurries were neutralized to pH 7 by the addition of 12 N HCl. Residual H_2_O_2_ was inactivated by addition of 59 μl of catalase (28 mg protein/ml; Sigma-Aldrich). After inactivation of catalase by heating at 90°C for 15 min, the flask contents were centrifuged and dried at 55°C. Alkaline peroxide treated material from transgenic lines 43A, 41A, 1A, 28ER, 24ER, 61 V, 15 V, 2 V and control were suspended at a final concentration of 0.5% (w/v) in 50 mM sodium citrate buffer (pH 5.0) containing 5 μg/ml tetracycline, 5 μg/ml cycloheximide and 0.02% sodium azide. The slurry was kept in suspension using a paddle reservoir designed for dispensing pharmaceutical beads on the Biomek FXP (model VP 756C-1P100; V&P Scientific, Inc., San Diego, CA, USA). A total of 200 μl of substrate slurry was then dispensed into mini-Eppendorf tubes, followed by addition of commercial enzymes (Accellerase 1500) at a final concentration of 15 mg protein per g of cellulose and the mixture was incubated at 50°C for 48 h. The tubes were centrifuged at 1,500 × *g* for 5 min to separate solid residue from hydrolyzed biomass. The supernatants (100 μl) were transferred into a Costar 96-well plate and heated at 100°C for 10 min to inactivate the enzymes. Each reaction mixture was run in duplicate, sampled twice, and the supernatants were assayed twice for released glucose using a K-GLUC kit (Megazyme, Bray, Ireland). Sugar assays were conducted in 96-well plates using 194 μl of assay reagent and 12 μl of sample. The plates were incubated at 50°C for 20 min before reading absorbance at 510 nm using the Synergy HT multi-detection microplate reader (Biotek Instruments, Inc.).

## Abbreviations

A: Apoplast; AHP: Alkaline hydrogen peroxide; AIR: Alcohol insoluble residue; ATR: Attenuated total reflectance; C: Control; CAZyme: Carbohydrate-active enzyme; CCAC: Canadian Council on Animal Care; CMC: Carboxymethylcellulose; DIG: Digoxigenin; EDTA: Ethylenediaminetetraacetic acid; ER: Endoplasmic reticulum; FAEA: Type A ferulic acid esterase; faeA: Feruloyl esterase A gene; FAEB: Type B ferulic acid esterase; faeB: Feruloyl esterase B gene; FTIR: Fourier transformed infrared spectroscopy; GC-MS: Gas chromatography–mass spectrometry; GUS: β-glucuronidase; IgG: Immunoglobulin G; IVDMD: *In vitro* dry matter disappearance; MOPS: 3-(N-morpholino)propanesulfonic acid; MSO: Murashige and Skoog medium; NPTII: Neomycin phosphotransferase II; OD: Optical density; PBS: Phosphate-buffered saline; PCA: Principal component analysis; PCR: Polymerase chain reaction; PIN: Potato proteinase inhibitor; PMSF: Phenylmethanesulfonyl fluoride; pNPG: *p*-Nitrophenyl-β-D-glucopyranoside; PVPP: Polyvinylpolypyrrolidone; Rubisco: Ribulose-1,5-bisphosphate carboxylase/oxygenase; TCA: Trichloroacetic acid; T-DNA: Transfer DNA; TFA: Trifluoroacetic acid; V: Vacuole; VFA: Volatile fatty acid.

## Competing interests

The authors declare that they have no competing interests.

## Authors’ contributions

DCWB, TM, AT, AB and BM conceived and designed the experiment. AB, LJ, SYH, KK, CJA and MLG performed the experiments. AB, LJ, SYH, KK, CJA, MLG, DCWB, TM and YW interpreted the data. AB, YW, TM, DCWB, AT, BM, LJ, SYH, KK, CJA and MLG drafted and revised the work. All authors read and approved the final manuscript.

## Supplementary Material

Additional file 1 Summary of alfalfa transformation experiments (n = 3). Click here for file

Additional file 2 **Southern blot analysis of *faeB*-apoplast line 10 A2.** The gDNA was cut with BamHI and the blot was probed with DIG-labeled *faeB* gene fragments (651 bp). Lane 1, 1 kb DNA ladder (1 μg); lane 2, wild type alfalfa gDNA (20 μg); lane 3, 10 A2 gDNA (20 μg); and lane 4, *faeB*-apoplast plasmid DNA (5 ng). Click here for file

Additional file 3 **Concentration of VFA (mM) after (A) 6 h and (B) 72 h of *in vitro *incubation of control and transgenic lines in mixed rumen fluid.** Bars indicate standard error. VFA, volatile fatty acid. Click here for file

Additional file 4 **Ammonia production during *in vitro *digestion of control and transgenic lines in mixed ruminal fluid.** Bars indicate standard error. *Differs to control at *P* < 0.05. Click here for file

Additional file 5 Correlation matrix table and correlation circle of axis F1 and F3. Click here for file

Additional file 6 **Difference between digestion of control and transgenic cell walls as evident from digital subtraction of FTIR spectra of respective digesta residue after 72 h of incubation with rumen fluid.** (A) Average spectrum of 24ER and 28ER; and (B) average spectrum of 43A, 41A and 1A versus wild type control. A, apoplast; ER, endoplasmic reticulum; FTIR, Fourier transformed infrared spectroscopy. Click here for file

Additional file 7 **FTIR spectral differences between control and transgenic cell walls after 6 h and 72 h of incubation with rumen fluid as an indicator of the progressive digestion of the plant cell wall.** (A) Wild type control; (B) average spectrum of 24ER and 28ER; and (C) average spectrum of 43A, 41A and 1A. A, apoplast; ER, endoplasmic reticulum; FTIR, Fourier transformed infrared spectroscopy. Click here for file

Additional file 8 **Total sugar and uronic acid content of cell walls.** (A) TFA and sulfuric acid solubilized total sugar content as determined by anthrone method. (B) Uronic acid content of TFA and sulfuric acid solubilized cell wall fractions. Bars indicate standard error of mean (n = 3). *Differs to control at *P* 0.05. Apoplast (A), *faeB*-apoplast (average of 43A, 41A and 1A); endoplasmic reticulum (ER), *faeB*-ER (average of 24ER and 28ER); and vacuole (V), *faeB-*vacuole (average of 61 V, 15 V and 2 V). A, apoplast; ER, endoplasmic reticulum; TFA, trifluoroacetic acid; V, vacuole; WT, wild type control. Click here for file

Additional file 9 **Schematic maps of vector sequence for apoplast, chloroplast, endoplasmic reticulum and vacuole targeted feruloyl esterase.** c-Myc, cMyc tag mouse antibody sequence from GenScript; CTPP, vacuole retention signal; ER, endoplasmic reticulum; KDEL, endoplasmic reticulum retention signal; PIN, potato protease inhibitor II terminator sequence; PR1b, secretory signal peptide from tobacco; StrepII, StrepII purification tag WSHPQFEK; tCUP4, enhanced tCUP4 promoter sequence. Click here for file

Additional file 10 **Sequences of synthetic *faeB *** **genes.** Click here for file

Additional file 11 **Schematic map of the 5,103 bp pEACH vector based on pPZP100**[36]. Modifications described in the Methods section. Par A, par A MYB recognition sequences. Click here for file

Additional file 12 Schematic map of the pEACH 7,205 construct showing restriction enzyme sites and insert size. Click here for file

Additional file 13 **Alfalfa tissue culture and transformation with *Agrobacterium tumefaciens *LBA4404.** (A) Callus formation on selection medium plates after culture with *Agrobacterium* containing *faeB* gene targeted four different cellular compartments (apoplast, chloroplast, vacuole and endoplasmic reticulum), 4 weeks. (B) Embryo formed on callus, 5 weeks. (C, D) Embryo formation, 7 weeks. (E, F) Embryo germination on selection medium, 9 to 10 weeks. (G) Transgenic alfalfa plants transferred to magenta box, 12 weeks. (H) Transgenic alfalfa plant transferred to soil, 18 weeks. (I) Potted transgenic alfalfa plants in a growth chamber, 28 weeks. Click here for file
