# Supplementary material for: Expression of a fungal ferulic acid esterase in alfalfa modifies cell wall digestibility
Source: Biotechnol Biofuels. 2014 Mar 20;7:39. doi: 10.1186/1754-6834-7-39 (PMC3999942; doi:10.1186/1754-6834-7-39)

**Additional file 2:** Southern blot analysis of FaeB-Apoplast line10A2. The gDNA was cut with BamHI, and the blot was probed with DIG-labelled FaeB gene fragments (651bp). Lane 1, 1 kb DNA ladder (1 μg); Lane 2, WT alfalfa gDNA (20 μg); Lane 3, 10 A2 gDNA (20 μg); FaeB-Apoplast plasmid DNA (5 ng).


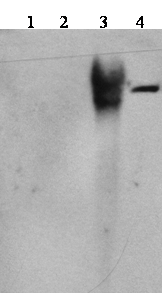

Supplement: Additional file 1 — Summary of alfalfa transformation experiments (n = 3). [file 1754-6834-7-39-S1.docx]
