# Supplementary material for: Expression of a fungal ferulic acid esterase in alfalfa modifies cell wall digestibility
Source: Biotechnol Biofuels. 2014 Mar 20;7:39. doi: 10.1186/1754-6834-7-39 (PMC3999942; doi:10.1186/1754-6834-7-39)

**Additional file 3:** Concentration of main volatile fatty acid (VFA; mM) after 6 h (A) and 72 h (B) of *in vitro* incubation of control and transgenic alfalfa lines with rumen fluid. Bars indicate standard error.


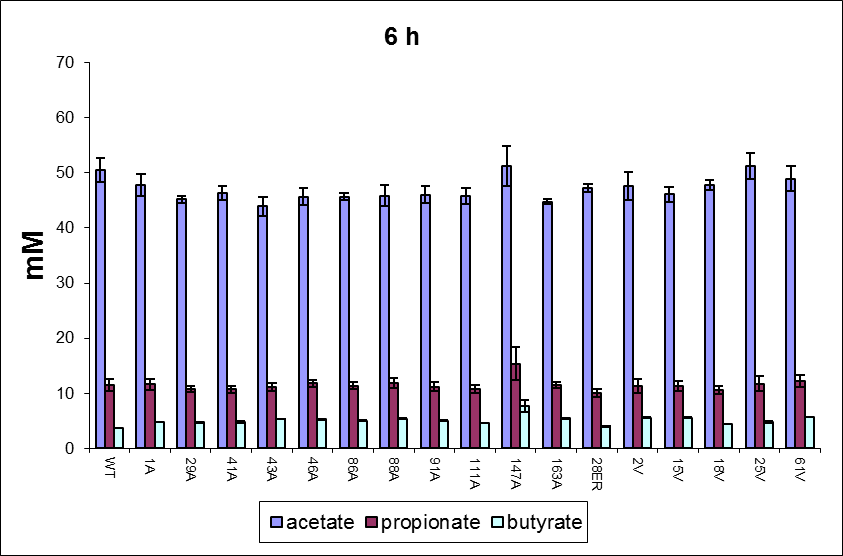

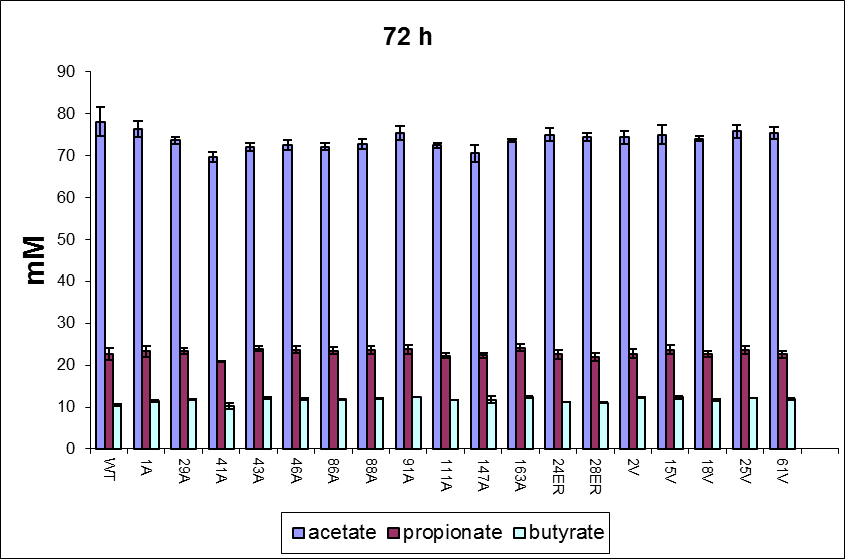

Supplement: Additional file 2 — Southern blot analysis of faeB-apoplast line 10 A2. The gDNA was cut with BamHI and the blot was probed with DIG-labeled faeB gene fragments (651 bp). Lane 1, 1 kb DNA ladder (1 μg); lane 2, wild type alfalfa gDNA (20 μg); lane 3, 10 A2 gDNA (20 μg); and lane 4, faeB-apoplast plasmid DNA (5 ng). [file 1754-6834-7-39-S2.docx]
