# Supplementary material for: Expression of a fungal ferulic acid esterase in alfalfa modifies cell wall digestibility
Source: Biotechnol Biofuels. 2014 Mar 20;7:39. doi: 10.1186/1754-6834-7-39 (PMC3999942; doi:10.1186/1754-6834-7-39)
Supplement: Additional file 3 — Concentration of VFA (mM) after (A) 6 h and (B) 72 h of in vitro incubation of control and transgenic lines in mixed rumen fluid. Bars indicate standard error. VFA, volatile fatty acid. [file 1754-6834-7-39-S3.docx]

**Additional file 5:** Correlation matrix table and correlation circle of axis F1vs F3.

| Pearson Correlation matrix (n): | | | |  |
| --- | --- | --- | --- | --- |
|  |  |  |  |  |
| Variables | control | 1a | er | 2v |
| control | 1 | 0.707 | 0.992 | 0.773 |
| 1a | 0.707 | 1 | 0.706 | 0.992 |
| er | 0.992 | 0.706 | 1 | 0.775 |
| 2v | 0.773 | 0.992 | 0.775 | 1 |

*All values differ from 0 at P=0.05*
