# Supplementary material for: Expression of a fungal ferulic acid esterase in alfalfa modifies cell wall digestibility
Source: Biotechnol Biofuels. 2014 Mar 20;7:39. doi: 10.1186/1754-6834-7-39 (PMC3999942; doi:10.1186/1754-6834-7-39)
Supplement: Additional file 4 — Ammonia production during in vitro digestion of control and transgenic lines in mixed ruminal fluid. Bars indicate standard error. *Differs to control at P < 0.05. [file 1754-6834-7-39-S4.docx]

**Additional file 6:** Difference between digestion of control and transgenic cell wall as evident from digital subtraction of FTIR spectra of respective digesta residue after 72 h of incubation with rumen fluid. Endoplasmic reticulum: Average spectra of lines 24ER and 28ER, Appoplast: Average spectra of lines 43A, 41A, 1A vs wild type control (W).


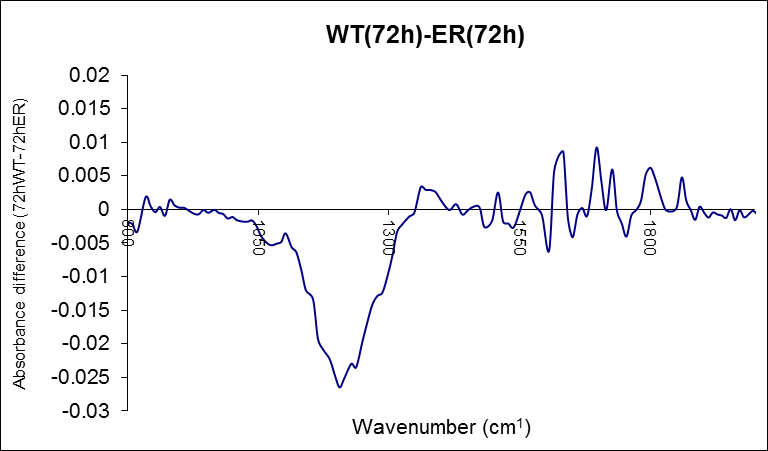


**A**

**Absorbance difference (72hW-72hER)**


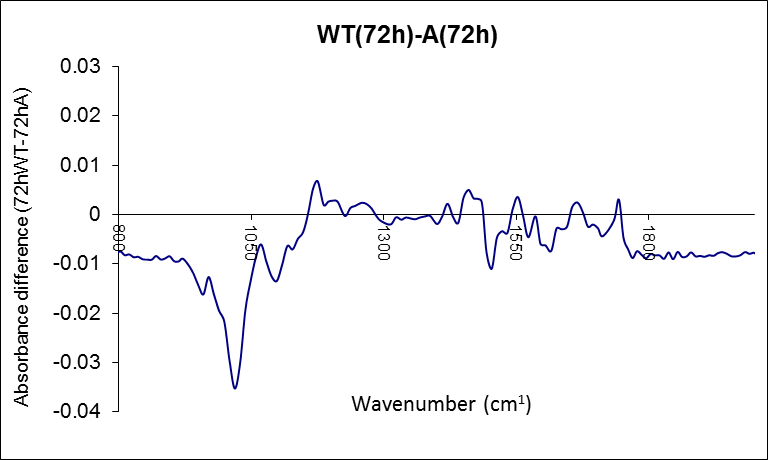


**Absorbance difference (72hW-72hA)**

**B**
