# Supplementary material for: Expression of a fungal ferulic acid esterase in alfalfa modifies cell wall digestibility
Source: Biotechnol Biofuels. 2014 Mar 20;7:39. doi: 10.1186/1754-6834-7-39 (PMC3999942; doi:10.1186/1754-6834-7-39)
Supplement: Additional file 5 — Correlation matrix table and correlation circle of axis F1 and F3. [file 1754-6834-7-39-S5.docx]

**Additional file 7:** FTIR spectral differences between control and transgenic lines after 6 and 72 h of incubation with rumen fluid as an indicator of the progressive digestion of the plant cell walls. Wild type control (WT); Endoplasmic reticulum (ER): Average spectra lines 24 ER and 28ER, Apoplast (A): Average spectra of 43A, 41A, 1A.


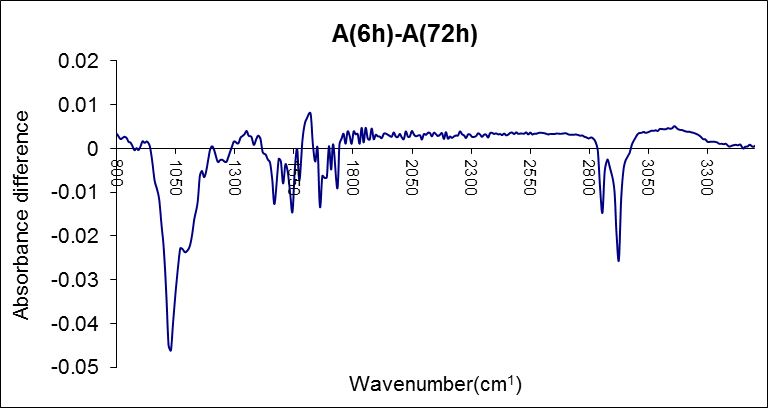
**
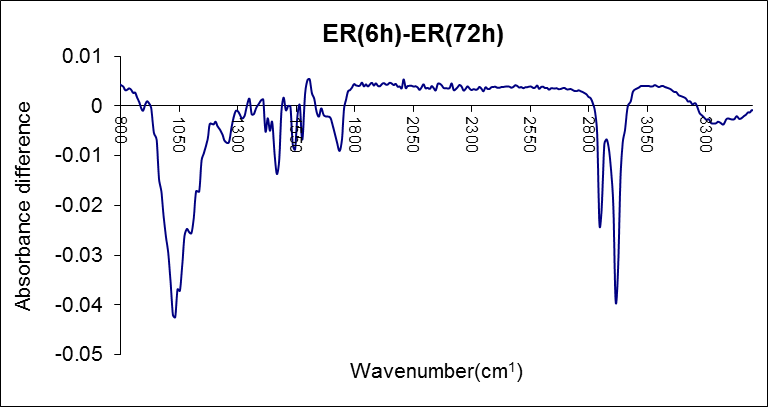
**
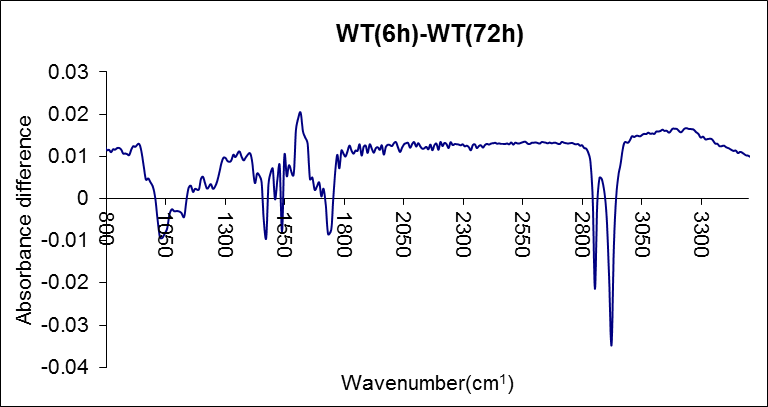


**C**

**B**

**A**

**Absorbance difference (6hA-72A)**

**Absorbance difference (6hER-72ER)**

**Absorbance difference (6hWT-72WT)**

**C**
