# Supplementary material for: Expression of a fungal ferulic acid esterase in alfalfa modifies cell wall digestibility
Source: Biotechnol Biofuels. 2014 Mar 20;7:39. doi: 10.1186/1754-6834-7-39 (PMC3999942; doi:10.1186/1754-6834-7-39)
Supplement: Additional file 6 — Difference between digestion of control and transgenic cell walls as evident from digital subtraction of FTIR spectra of respective digesta residue after 72 h of incubation with rumen fluid. (A) Average spectrum of 24ER and 28ER; and (B) average spectrum of 43A, 41A and 1A versus wild type control. A, apoplast; ER, endoplasmic reticulum; FTIR, Fourier transformed infrared spectroscopy. [file 1754-6834-7-39-S6.docx]

**Additional file 8**: A) Trifluoroacetic (TFA) and sulphuric acid solubilized total sugar content as determined by anthrone method. B) Uronic acid content of trifluoroacetic and sulphuric acid solubilized cell wall fractions. Bars indicate standard errors of mean (n=3). (*) differs to control at (p˂0.05). WT, wild type; A, *Fa*eB–apoplast (Average of 43A, 41A, 1A); ER, *Fa*eB–endoplasmic reticulum (Average of 24 ER and 28ER); V, *Fa*eB –vacuole (Average of 61V, 15V, 2V).
