# Supplementary material for: Expression of a fungal ferulic acid esterase in alfalfa modifies cell wall digestibility
Source: Biotechnol Biofuels. 2014 Mar 20;7:39. doi: 10.1186/1754-6834-7-39 (PMC3999942; doi:10.1186/1754-6834-7-39)
Supplement: Additional file 7 — FTIR spectral differences between control and transgenic cell walls after 6 h and 72 h of incubation with rumen fluid as an indicator of the progressive digestion of the plant cell wall. (A) Wild type control; (B) average spectrum of 24ER and 28ER; and (C) average spectrum of 43A, 41A and 1A. A, apoplast; ER, endoplasmic reticulum; FTIR, Fourier transformed infrared spectroscopy. [file 1754-6834-7-39-S7.docx]

**Additional file 9:** Schematic maps of vector sequence for apoplast, chloroplast, endoplasmic reticulum and vacuole targeted Ferolyl esterase. tCUP4, enhanced tCUp 4 promoter sequence; PR1b, secretory signal peptide from tobacco; c-myc, cMyc tag mouse antibody sequence from GenScript; StrepII, strepII purification tag WSHPQFEK; PIN, potato protease inhibitor II terminator sequence; CTPP vacuole retention signal; KDEL Endoplasmic reticulum retention signal.
