# Supplementary material for: Expression of a fungal ferulic acid esterase in alfalfa modifies cell wall digestibility
Source: Biotechnol Biofuels. 2014 Mar 20;7:39. doi: 10.1186/1754-6834-7-39 (PMC3999942; doi:10.1186/1754-6834-7-39)
Supplement: Additional file 8 — Total sugar and uronic acid content of cell walls. (A) TFA and sulfuric acid solubilized total sugar content as determined by anthrone method. (B) Uronic acid content of TFA and sulfuric acid solubilized cell wall fractions. Bars indicate standard error of mean (n = 3). *Differs to control at P 0.05. Apoplast (A), faeB-apoplast (average of 43A, 41A and 1A); endoplasmic reticulum (ER), faeB-ER (average of 24ER and 28ER); and vacuole (V), faeB-vacuole (average of 61 V, 15 V and 2 V). A, apoplast; ER, endoplasmic reticulum; TFA, trifluoroacetic acid; V, vacuole; WT, wild type control. [file 1754-6834-7-39-S8.docx]

**Additional file 10:** Sequences of synthetic *Fae*B genes.

*Fae*B-Apoplast:

***Xba***I 10 20 30 40 50 60

1 TCTAGAATGGGTTTCTTTTTGTTTTCTCAAATGCCTTCATTTTTCTTGGTTTCTACACTT

1 S R M G F F L F S Q M P S F F L V S T L

**PR1b**

70 80 90 100 110 120

61 TTGCTTTTCCTTATTATTTCACATTCTTCACATGCTACTGATCCATTTCAAAGTAGATGT

21 L L F L I I S H S S H A T D P F Q S R C

130 140 150 160 170 180

121 AATGAGTTTCAAAACAAAATTGATATTGCTAATGTTACTGTTAGGTCTGTTGCATATGTT

41 N E F Q N K I D I A N V T V R S V A Y V

190 200 210 220 230 240

181 GCTGCTGGTCAAAACATTTCTCAAGCTGAGGTTGCATCAGTTTGTAAGGCTAGTGTTCAA

61 A A G Q N I S Q A E V A S V C K A S V Q

250 260 270 280 290 300

241 GCATCTGTTGATCTTTGTAGGGTTACTATGAATATTTCTACATCAGATCGTTCTCATTTG

81 A S V D L C R V T M N I S T S D R S H L

310 320 330 340 350 360

301 TGGGCTGAAGCATGGCTTCCTAGAAATTATACTGGAAGGTTTGTTTCAACAGGAAACGGT

101 W A E A W L P R N Y T G R F V S T G N G

370 380 390 400 410 420

361 GGATTGGCTGGTTGTGTTCAAGAGACAGATCTTAATTTCGCTGCAAACTTTGGTTTCGCT

121 G L A G C V Q E T D L N F A A N F G F A

430 440 450 460 470 480

421 ACTGTTGGAACAAATGGTGGACATGATGGAGATACTGCAAAGTATTTTCTTAATAACAGT

141 T V G T N G G H D G D T A K Y F L N N S

490 500 510 520 530 540

481 GAAGTTCTTGCTGATTTCGCATACAGATCTGTTCATGAGGGTACAGTTGTTGGAAAGCAA

161 E V L A D F A Y R S V H E G T V V G K Q

550 560 570 580 590 600

541 TTGACTCAATTGTTTTATGATGAAGGATACAACTACAGTTACTACTTGGGTTGTTCTACA

181 L T Q L F Y D E G Y N Y S Y Y L G C S T

610 620 630 640 650 660

601 GGTGGACGTCAAGGATATCAACAAGTTCAAAGATTCCCTGATGATTACGATGGTGTTATT

201 G G R Q G Y Q Q V Q R F P D D Y D G V I

670 680 690 700 710 720

661 GCTGGTTCTGCTGCTATGAATTTTATTAACTTGATTAGTTGGGGTGCTTTCTTGTGGAAA

221 A G S A A M N F I N L I S W G A F L W K

730 740 750 760 770 780

721 GCTACTGGACTTGCAGATGATCCAGATTTCATTTCAGCTAATCTTTGGAGTGTTATTCAT

241 A T G L A D D P D F I S A N L W S V I H

790 800 810 820 830 840

781 CAAGAAATTGTTAGGCAATGTGATTTGGTTGATGGTGCTCTTGATGGAATTATTGAAGAT

261 Q E I V R Q C D L V D G A L D G I I E D

850 860 870 880 890 900

841 CCTGATTTCTGTGCACCAGTTATTGAGCGTTTGATTTGTGATGGTACTACAAATGGTACT

281 P D F C A P V I E R L I C D G T T N G T

910 920 930 940 950 960

901 TCTTGTATTACAGGTGCTCAAGCTGCAAAGGTTAACCGTGCACTTTCAGATTTTTATGGT

301 S C I T G A Q A A K V N R A L S D F Y G

970 980 990 1000 1010 1020

961 CCTGATGGAACAGTTTATTACCCAAGATTGAATTACGGTGGAGAGGCTGATAGTGCATCT

321 P D G T V Y Y P R L N Y G G E A D S A S

1030 1040 1050 1060 1070 1080

1021 CTTTATTTCACAGGATCAATGTACAGTAGGACTGAAGAGTGGTATAAGTATGTTGTTTAC

341 L Y F T G S M Y S R T E E W Y K Y V V Y

1090 1100 1110 1120 1130 1140

1081 AACGATACAAACTGGAACAGTTCTCAGTGGACTCTTGAATCTGCTAAGTTGGCACTTGAG

361 N D T N W N S S Q W T L E S A K L A L E

1150 1160 1170 1180 1190 1200

1141 CAAAACCCTTTTAACATTCAAGCATTTGATCCAAACATTACTGCATTTCGTGATAGAGGT

381 Q N P F N I Q A F D P N I T A F R D R G

1210 1220 1230 1240 1250 1260

1201 GGAAAATTGCTTTCATATCATGGTACTCAAGATCCTATTATTTCAAGTACTGATTCTAAA

401 G K L L S Y H G T Q D P I I S S T D S K

1270 1280 1290 1300 1310 1320

1261 CTTTATTACAGAAGGGTTGCTAATGCATTGAACGCTGCACCAAGTGAACTTGATGAGTTT

421 L Y Y R R V A N A L N A A P S E L D E F

1330 1340 1350 1360 1370 1380

1321 TACAGATTTTTCCAAATTTCTGGTATGGGACATTGTGGAGATGGAACAGGTGCTTCTTAC

441 Y R F F Q I S G M G H C G D G T G A S Y

1390 1400 1410 1420 1430 1440

1381 ATTGGACAAGGTTATGGAACTTACACATCAAAAGCACCACAAGTTAATTTGCTTAGGACT

461 I G Q G Y G T Y T S K A P Q V N L L R T

1450 1460 1470 1480 1490 1500

1441 ATGGTTGATTGGGTTGAAAATGGTAAAGCTCCTGAGTATATGCCAGGAAATAAGTTGAAT

481 M V D W V E N G K A P E Y M P G N K L N

1510 1520 1530 1540 1550 1560

1501 GCAAACGGTTCTATTGAATACATGAGGAAGCATTGTCGTTACCCTAAGCATAACATTCAT

501 A N G S I E Y M R K H C R Y P K H N I H

1570 1580 1590 1600 1610 1620

1561 ACTGGTCCTGGAAATTACACAGATCCAAACTCATGGACTTGTGTTGAACAAAAATTGATT

521 T G P G N Y T D P N S W T C V E Q K L I

1630 1640 1650 1660 *Bam*HI

1621 TCAGAAGAGGATCTTTGGAGTCATCCACAATTTGAGAAGTAAGGATCC

541 S E E D L W S H P Q F E K * G S

**c-myc strep II**

*Fae*B-Chloroplast:

***Xba***I 10 20 30 40 50 60

1 TCTAGAATGGCTTCTTCAGTTCTTTCTTCTGCTGCTGTTGCAACAAGGTCTAATGTTGCT

1 S R M A S S V L S S A A V A T R S N V A

70 80 90 100 110 120

61 CAAGCAAACATGGTTGCTCCTTTTACTGGTCTTAAATCTGCTGCATCATTCCCAGTTTCA

21 Q A N M V A P F T G L K S A A S F P V S

**Rubisco leading peptides**

130 140 150 160 170 180

121 CGTAAACAAAATTTGGATATTACAAGTATTGCTTCTAACGGTGGAAGGGTTCAATGTACT

41 R K Q N L D I T S I A S N G G R V Q C T

190 200 210 220 230 240

181 GATCCTTTCCAATCACGTTGTAATGAGTTTCAAAACAAAATTGATATTGCTAATGTTACT

61 D P F Q S R C N E F Q N K I D I A N V T

250 260 270 280 290 300

241 GTTAGAAGTGTTGCATATGTTGCTGCTGGTCAAAACATTTCACAAGCTGAGGTTGCAAGT

81 V R S V A Y V A A G Q N I S Q A E V A S

310 320 330 340 350 360

301 GTTTGTAAGGCTTCTGTTCAAGCATCAGTTGATTTGTGTAGAGTTACTATGAATATTAGT

101 V C K A S V Q A S V D L C R V T M N I S

370 380 390 400 410 420

361 ACATCTGATAGGTCTCATCTTTGGGCTGAAGCATGGTTGCCAAGAAATTATACTGGAAGG

121 T S D R S H L W A E A W L P R N Y T G R

430 440 450 460 470 480

421 TTTGTTTCAACAGGAAACGGTGGACTTGCTGGTTGTGTTCAAGAGACAGATTTGAATTTC

141 F V S T G N G G L A G C V Q E T D L N F

490 500 510 520 530 540

481 GCTGCAAACTTTGGTTTCGCTACTGTTGGAACAAATGGTGGACATGATGGAGATACTGCA

161 A A N F G F A T V G T N G G H D G D T A

550 560 570 580 590 600

541 AAGTATTTTCTTAATAACTCAGAAGTTTTGGCTGATTTCGCATACAGAAGTGTTCATGAG

181 K Y F L N N S E V L A D F A Y R S V H E

610 620 630 640 650 660

601 GGTACAGTTGTTGGAAAGCAACTTACTCAATTGTTTTATGATGAAGGATACAACTACTCT

201 G T V V G K Q L T Q L F Y D E G Y N Y S

670 680 690 700 710 720

661 TACTACCTTGGTTGTTCAACAGGTGGACGTCAAGGATATCAACAAGTTCAAAGATTCCCT

221 Y Y L G C S T G G R Q G Y Q Q V Q R F P

730 740 750 760 770 780

721 GATGATTACGATGGTGTTATTGCTGGTTCTGCTGCTATGAATTTTATTAACCTTATTTCA

241 D D Y D G V I A G S A A M N F I N L I S

790 800 810 820 830 840

781 TGGGGTGCTTTCCTTTGGAAAGCTACTGGATTGGCAGATGATCCAGATTTCATTAGTGCT

261 W G A F L W K A T G L A D D P D F I S A

850 860 870 880 890 900

841 AATTTGTGGTCTGTTATTCATCAAGAAATTGTTAGACAATGTGATCTTGTTGATGGTGCT

281 N L W S V I H Q E I V R Q C D L V D G A

910 920 930 940 950 960

901 TTGGATGGAATTATTGAAGATCCTGATTTCTGTGCACCAGTTATTGAGAGGCTTATTTGT

301 L D G I I E D P D F C A P V I E R L I C

970 980 990 1000 1010 1020

961 GATGGTACTACAAATGGTACTTCTTGTATTACAGGTGCTCAAGCTGCAAAGGTTAACCGT

321 D G T T N G T S C I T G A Q A A K V N R

1030 1040 1050 1060 1070 1080

1021 GCATTGTCTGATTTTTATGGTCCTGATGGAACAGTTTATTACCCAAGACTTAATTACGGT

341 A L S D F Y G P D G T V Y Y P R L N Y G

1090 1100 1110 1120 1130 1140

1081 GGAGAGGCTGATTCAGCAAGTTTGTATTTCACAGGATCAATGTACAGTAGAACTGAAGAG

361 G E A D S A S L Y F T G S M Y S R T E E

1150 1160 1170 1180 1190 1200

1141 TGGTATAAGTATGTTGTTTACAACGATACAAACTGGAACTCAAGTCAGTGGACTCTTGAA

381 W Y K Y V V Y N D T N W N S S Q W T L E

1210 1220 1230 1240 1250 1260

1201 TCTGCTAAACTTGCATTGGAGCAAAACCCTTTTAACATTCAAGCATTTGATCCAAACATT

401 S A K L A L E Q N P F N I Q A F D P N I

1270 1280 1290 1300 1310 1320

1261 ACTGCATTTCGTGATAGAGGTGGAAAACTTTTGTCTTATCATGGTACTCAAGATCCTATT

421 T A F R D R G G K L L S Y H G T Q D P I

1330 1340 1350 1360 1370 1380

1321 ATTTCTTCAACTGATTCAAAGTTGTATTACAGAAGGGTTGCTAATGCACTTAACGCTGCA

441 I S S T D S K L Y Y R R V A N A L N A A

1390 1400 1410 1420 1430 1440

1381 CCATCAGAATTGGATGAGTTTTACAGATTTTTCCAAATTAGTGGTATGGGACATTGTGGA

461 P S E L D E F Y R F F Q I S G M G H C G

1450 1460 1470 1480 1490 1500

1441 GATGGAACAGGTGCTAGTTACATTGGACAAGGTTATGGAACTTACACATCTAAAGCACCT

481 D G T G A S Y I G Q G Y G T Y T S K A P

1510 1520 1530 1540 1550 1560

1501 CAAGTTAATCTTTTGAGGACTATGGTTGATTGGGTTGAAAATGGTAAAGCTCCTGAGTAT

501 Q V N L L R T M V D W V E N G K A P E Y

1570 1580 1590 1600 1610 1620

1561 ATGCCAGGAAATAAGTTGAATGCAAACGGTTCTATTGAATACATGAGGAAGCATTGTCGT

521 M P G N K L N A N G S I E Y M R K H C R

1630 1640 1650 1660 1670 1680

1621 TACCCTAAGCATAACATTCATACTGGTCCTGGAAATTACACAGATCCAAACTCATGGACT

541 Y P K H N I H T G P G N Y T D P N S W T

1690 1700 1710 1720 1730 1740

1681 TGTGTTGAACAAAAACTTATTAGTGAAGAGGATTTGTGGTCTCATCCACAATTTGAGAAG

561 C V E Q K L I S E E D L W S H P Q F E K

**c-myc strepII**

***Bam*H**I

1741 TAAGGATCC

581 * G S

*Fae*B-ER:

***Xba*I** 10 20 30 40 50 60

1 TCTAGAATGGGTTTCTTTTTGTTTTCTCAAATGCCTTCATTTTTCTTGGTTTCTACACTT

1 S R M G F F L F S Q M P S F F L V S T L

**PR1b**

70 80 90 100 110 120

61 TTGCTTTTCCTTATTATTTCACATTCTTCACATGCTACTGATCCATTTCAAAGTAGATGT

21 L L F L I I S H S S H A T D P F Q S R C

130 140 150 160 170 180

121 AATGAGTTTCAAAACAAAATTGATATTGCTAATGTTACTGTTAGGTCTGTTGCATATGTT

41 N E F Q N K I D I A N V T V R S V A Y V

190 200 210 220 230 240

181 GCTGCTGGTCAAAACATTTCTCAAGCTGAGGTTGCATCAGTTTGTAAGGCTAGTGTTCAA

61 A A G Q N I S Q A E V A S V C K A S V Q

250 260 270 280 290 300

241 GCATCTGTTGATCTTTGTAGGGTTACTATGAATATTTCTACATCAGATCGTTCTCATTTG

81 A S V D L C R V T M N I S T S D R S H L

310 320 330 340 350 360

301 TGGGCTGAAGCATGGCTTCCTAGAAATTATACTGGAAGGTTTGTTTCAACAGGAAACGGT

101 W A E A W L P R N Y T G R F V S T G N G

370 380 390 400 410 420

361 GGATTGGCTGGTTGTGTTCAAGAGACAGATCTTAATTTCGCTGCAAACTTTGGTTTCGCT

121 G L A G C V Q E T D L N F A A N F G F A

430 440 450 460 470 480

421 ACTGTTGGAACAAATGGTGGACATGATGGAGATACTGCAAAGTATTTTCTTAATAACAGT

141 T V G T N G G H D G D T A K Y F L N N S

490 500 510 520 530 540

481 GAAGTTCTTGCTGATTTCGCATACAGATCTGTTCATGAGGGTACAGTTGTTGGAAAGCAA

161 E V L A D F A Y R S V H E G T V V G K Q

550 560 570 580 590 600

541 TTGACTCAATTGTTTTATGATGAAGGATACAACTACAGTTACTACTTGGGTTGTTCTACA

181 L T Q L F Y D E G Y N Y S Y Y L G C S T

610 620 630 640 650 660

601 GGTGGACGTCAAGGATATCAACAAGTTCAAAGATTCCCTGATGATTACGATGGTGTTATT

201 G G R Q G Y Q Q V Q R F P D D Y D G V I

670 680 690 700 710 720

661 GCTGGTTCTGCTGCTATGAATTTTATTAACTTGATTAGTTGGGGTGCTTTCTTGTGGAAA

221 A G S A A M N F I N L I S W G A F L W K

730 740 750 760 770 780

721 GCTACTGGACTTGCAGATGATCCAGATTTCATTTCAGCTAATCTTTGGAGTGTTATTCAT

241 A T G L A D D P D F I S A N L W S V I H

790 800 810 820 830 840

781 CAAGAAATTGTTAGGCAATGTGATTTGGTTGATGGTGCTCTTGATGGAATTATTGAAGAT

261 Q E I V R Q C D L V D G A L D G I I E D

850 860 870 880 890 900

841 CCTGATTTCTGTGCACCAGTTATTGAGCGTTTGATTTGTGATGGTACTACAAATGGTACT

281 P D F C A P V I E R L I C D G T T N G T

910 920 930 940 950 960

901 TCTTGTATTACAGGTGCTCAAGCTGCAAAGGTTAACCGTGCACTTTCAGATTTTTATGGT

301 S C I T G A Q A A K V N R A L S D F Y G

970 980 990 1000 1010 1020

961 CCTGATGGAACAGTTTATTACCCAAGATTGAATTACGGTGGAGAGGCTGATAGTGCATCT

321 P D G T V Y Y P R L N Y G G E A D S A S

1030 1040 1050 1060 1070 1080

1021 CTTTATTTCACAGGATCAATGTACAGTAGGACTGAAGAGTGGTATAAGTATGTTGTTTAC

341 L Y F T G S M Y S R T E E W Y K Y V V Y

1090 1100 1110 1120 1130 1140

1081 AACGATACAAACTGGAACAGTTCTCAGTGGACTCTTGAATCTGCTAAGTTGGCACTTGAG

361 N D T N W N S S Q W T L E S A K L A L E

1150 1160 1170 1180 1190 1200

1141 CAAAACCCTTTTAACATTCAAGCATTTGATCCAAACATTACTGCATTTCGTGATAGAGGT

381 Q N P F N I Q A F D P N I T A F R D R G

1210 1220 1230 1240 1250 1260

1201 GGAAAATTGCTTTCATATCATGGTACTCAAGATCCTATTATTTCAAGTACTGATTCTAAA

401 G K L L S Y H G T Q D P I I S S T D S K

1270 1280 1290 1300 1310 1320

1261 CTTTATTACAGAAGGGTTGCTAATGCATTGAACGCTGCACCAAGTGAACTTGATGAGTTT

421 L Y Y R R V A N A L N A A P S E L D E F

1330 1340 1350 1360 1370 1380

1321 TACAGATTTTTCCAAATTTCTGGTATGGGACATTGTGGAGATGGAACAGGTGCTTCTTAC

441 Y R F F Q I S G M G H C G D G T G A S Y

1390 1400 1410 1420 1430 1440

1381 ATTGGACAAGGTTATGGAACTTACACATCAAAAGCACCACAAGTTAATTTGCTTAGGACT

461 I G Q G Y G T Y T S K A P Q V N L L R T

1450 1460 1470 1480 1490 1500

1441 ATGGTTGATTGGGTTGAAAATGGTAAAGCTCCTGAGTATATGCCAGGAAATAAGTTGAAT

481 M V D W V E N G K A P E Y M P G N K L N

1510 1520 1530 1540 1550 1560

1501 GCAAACGGTTCTATTGAATACATGAGGAAGCATTGTCGTTACCCTAAGCATAACATTCAT

501 A N G S I E Y M R K H C R Y P K H N I H

1570 1580 1590 1600 1610 1620

1561 ACTGGTCCTGGAAATTACACAGATCCAAACTCATGGACTTGTGTTGAACAAAAATTGATT

521 T G P G N Y T D P N S W T C V E Q K L I

1630 1640 1650 1660 1670 1680

1621 TCAGAAGAGGATCTTTGGAGTCATCCACAATTTGAGAAGAAAGATGAACTTTAAGGATCC

541 S E E D L W S H P Q F E K K D E L * G S

***Bam*HI**

c-myc strepII KDEL

*Fae*B-Vacuole:

***Xba*I** 10 20 30 40 50 60

1 TCTAGAATGGGTTTCTTTTTGTTTTCTCAAATGCCTTCATTTTTCTTGGTTTCTACACTT

1 S R M G F F L F S Q M P S F F L V S T L

**PR1b**

70 80 90 100 110 120

61 TTGCTTTTCCTTATTATTTCACATTCTTCACATGCTACTGATCCATTTCAAAGTAGATGT

21 L L F L I I S H S S H A T D P F Q S R C

130 140 150 160 170 180

121 AATGAGTTTCAAAACAAAATTGATATTGCTAATGTTACTGTTAGGTCTGTTGCATATGTT

41 N E F Q N K I D I A N V T V R S V A Y V

190 200 210 220 230 240

181 GCTGCTGGTCAAAACATTTCTCAAGCTGAGGTTGCATCAGTTTGTAAGGCTAGTGTTCAA

61 A A G Q N I S Q A E V A S V C K A S V Q

250 260 270 280 290 300

241 GCATCTGTTGATCTTTGTAGGGTTACTATGAATATTTCTACATCAGATCGTTCTCATTTG

81 A S V D L C R V T M N I S T S D R S H L

310 320 330 340 350 360

301 TGGGCTGAAGCATGGCTTCCTAGAAATTATACTGGAAGGTTTGTTTCAACAGGAAACGGT

101 W A E A W L P R N Y T G R F V S T G N G

370 380 390 400 410 420

361 GGATTGGCTGGTTGTGTTCAAGAGACAGATCTTAATTTCGCTGCAAACTTTGGTTTCGCT

121 G L A G C V Q E T D L N F A A N F G F A

430 440 450 460 470 480

421 ACTGTTGGAACAAATGGTGGACATGATGGAGATACTGCAAAGTATTTTCTTAATAACAGT

141 T V G T N G G H D G D T A K Y F L N N S

490 500 510 520 530 540

481 GAAGTTCTTGCTGATTTCGCATACAGATCTGTTCATGAGGGTACAGTTGTTGGAAAGCAA

161 E V L A D F A Y R S V H E G T V V G K Q

550 560 570 580 590 600

541 TTGACTCAATTGTTTTATGATGAAGGATACAACTACAGTTACTACTTGGGTTGTTCTACA

181 L T Q L F Y D E G Y N Y S Y Y L G C S T

610 620 630 640 650 660

601 GGTGGACGTCAAGGATATCAACAAGTTCAAAGATTCCCTGATGATTACGATGGTGTTATT

201 G G R Q G Y Q Q V Q R F P D D Y D G V I

670 680 690 700 710 720

661 GCTGGTTCTGCTGCTATGAATTTTATTAACTTGATTAGTTGGGGTGCTTTCTTGTGGAAA

221 A G S A A M N F I N L I S W G A F L W K

730 740 750 760 770 780

721 GCTACTGGACTTGCAGATGATCCAGATTTCATTTCAGCTAATCTTTGGAGTGTTATTCAT

241 A T G L A D D P D F I S A N L W S V I H

790 800 810 820 830 840

781 CAAGAAATTGTTAGGCAATGTGATTTGGTTGATGGTGCTCTTGATGGAATTATTGAAGAT

261 Q E I V R Q C D L V D G A L D G I I E D

850 860 870 880 890 900

841 CCTGATTTCTGTGCACCAGTTATTGAGCGTTTGATTTGTGATGGTACTACAAATGGTACT

281 P D F C A P V I E R L I C D G T T N G T

910 920 930 940 950 960

901 TCTTGTATTACAGGTGCTCAAGCTGCAAAGGTTAACCGTGCACTTTCAGATTTTTATGGT

301 S C I T G A Q A A K V N R A L S D F Y G

970 980 990 1000 1010 1020

961 CCTGATGGAACAGTTTATTACCCAAGATTGAATTACGGTGGAGAGGCTGATAGTGCATCT

321 P D G T V Y Y P R L N Y G G E A D S A S

1030 1040 1050 1060 1070 1080

1021 CTTTATTTCACAGGATCAATGTACAGTAGGACTGAAGAGTGGTATAAGTATGTTGTTTAC

341 L Y F T G S M Y S R T E E W Y K Y V V Y

1090 1100 1110 1120 1130 1140

1081 AACGATACAAACTGGAACAGTTCTCAGTGGACTCTTGAATCTGCTAAGTTGGCACTTGAG

361 N D T N W N S S Q W T L E S A K L A L E

1150 1160 1170 1180 1190 1200

1141 CAAAACCCTTTTAACATTCAAGCATTTGATCCAAACATTACTGCATTTCGTGATAGAGGT

381 Q N P F N I Q A F D P N I T A F R D R G

1210 1220 1230 1240 1250 1260

1201 GGAAAATTGCTTTCATATCATGGTACTCAAGATCCTATTATTTCAAGTACTGATTCTAAA

401 G K L L S Y H G T Q D P I I S S T D S K

1270 1280 1290 1300 1310 1320

1261 CTTTATTACAGAAGGGTTGCTAATGCATTGAACGCTGCACCAAGTGAACTTGATGAGTTT

421 L Y Y R R V A N A L N A A P S E L D E F

1330 1340 1350 1360 1370 1380

1321 TACAGATTTTTCCAAATTTCTGGTATGGGACATTGTGGAGATGGAACAGGTGCTTCTTAC

441 Y R F F Q I S G M G H C G D G T G A S Y

1390 1400 1410 1420 1430 1440

1381 ATTGGACAAGGTTATGGAACTTACACATCAAAAGCACCACAAGTTAATTTGCTTAGGACT

461 I G Q G Y G T Y T S K A P Q V N L L R T

1450 1460 1470 1480 1490 1500

1441 ATGGTTGATTGGGTTGAAAATGGTAAAGCTCCTGAGTATATGCCAGGAAATAAGTTGAAT

481 M V D W V E N G K A P E Y M P G N K L N

1510 1520 1530 1540 1550 1560

1501 GCAAACGGTTCTATTGAATACATGAGGAAGCATTGTCGTTACCCTAAGCATAACATTCAT

501 A N G S I E Y M R K H C R Y P K H N I H

1570 1580 1590 1600 1610 1620

1561 ACTGGTCCTGGAAATTACACAGATCCAAACTCATGGACTTGTGTTGAACAAAAATTGATT

521 T G P G N Y T D P N S W T C V E Q K L I

1630 1640 1650 1660 1670 1680

1621 TCAGAAGAGGATCTTTGGAGTCATCCACAATTTGAGAAGAACGGTTTGCTTGTTGATACT

541 S E E D L W S H P Q F E K N G L L V D T

c-myc strepII CTPP

1690

1681 ATGTAAGGATCC

561 M * G S
