# Supplementary material for: Expression of a fungal ferulic acid esterase in alfalfa modifies cell wall digestibility
Source: Biotechnol Biofuels. 2014 Mar 20;7:39. doi: 10.1186/1754-6834-7-39 (PMC3999942; doi:10.1186/1754-6834-7-39)
Supplement: Additional file 9 — Schematic maps of vector sequence for apoplast, chloroplast, endoplasmic reticulum and vacuole targeted feruloyl esterase. c-Myc, cMyc tag mouse antibody sequence from GenScript; CTPP, vacuole retention signal; ER, endoplasmic reticulum; KDEL, endoplasmic reticulum retention signal; PIN, potato protease inhibitor II terminator sequence; PR1b, secretory signal peptide from tobacco; StrepII, StrepII purification tag WSHPQFEK; tCUP4, enhanced tCUP4 promoter sequence. [file 1754-6834-7-39-S9.docx]

**Additional file 11:** Schematic map of the 5103 bp pEACH vector based on pZIP100 (Hadjdukiewics et al., 1994). Modifications are described in the methods section. Par A, par A MRS recognition sequences.

pPEACH 5103

**250bp**

**850bp**

**515bp**

**410bp**

**2067bp**

**515bp**

**nptII**

**PIN**

**GUSintron**

**tCUP4**

**ARBC**

**tCUP4**

**filler**

**LB**

**vector**

**vector**

**RB**

**filler**

**XbaI, ApaI**

**XhoI**

**NcoI, BamHI, Xbal**

**PstI, SalI, EcoRI, KpnI**

**BamHI**

**XbaI**

**PstI, HindIII, KpnI, SmaI, SacI, Xhol**
