# Supplementary material for: Expression of a fungal ferulic acid esterase in alfalfa modifies cell wall digestibility
Source: Biotechnol Biofuels. 2014 Mar 20;7:39. doi: 10.1186/1754-6834-7-39 (PMC3999942; doi:10.1186/1754-6834-7-39)

**Additional file 12:** Schematic map of the pEACH 7205 construct showing restriction enzyme sites and insert size. LB, T-DNA left border; RB, T-DNA right border; ARBC;Arabidopsis rbcS terminator; nptII, kanamycin; GUS intron, beta-glucuronidase with a catalase intron; PIN, potato protease inhibitor II terminator sequence; tCUP4, enhanced tCUp 4 promoter sequence.


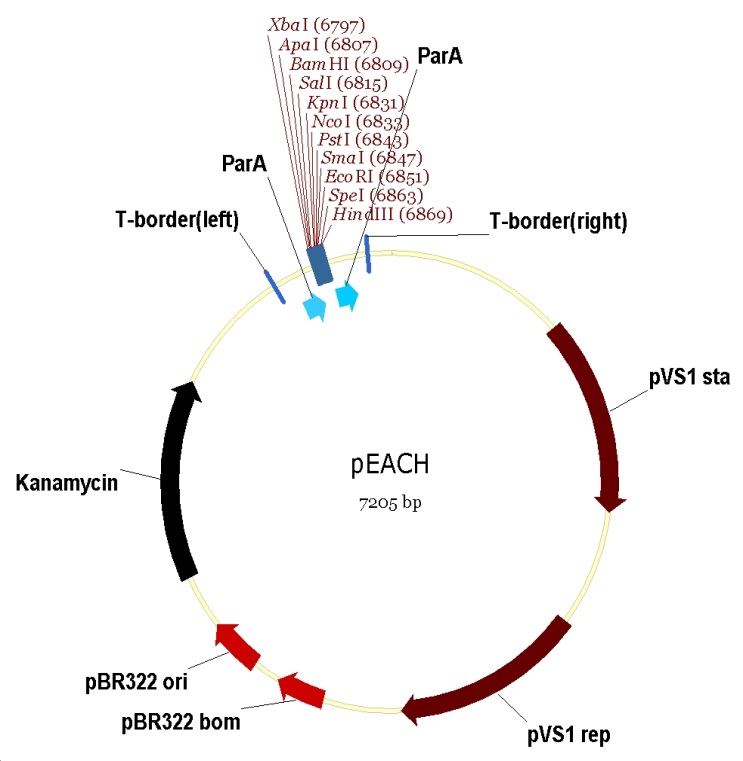

Supplement: Additional file 10 — Sequences of synthetic faeB genes. [file 1754-6834-7-39-S10.docx]
