# Supplementary material for: Expression of a fungal ferulic acid esterase in alfalfa modifies cell wall digestibility
Source: Biotechnol Biofuels. 2014 Mar 20;7:39. doi: 10.1186/1754-6834-7-39 (PMC3999942; doi:10.1186/1754-6834-7-39)

**Additional file 13:** Alfalfa tissue culture and transformation with *Agrobacterium tumefaciens* LBA4404: (A) Callus formation on selection medium plates after-culture with *Agrobacterium* containing FaeB gene targeted four different cellular compartments (apoplast, chloroplast, vacuole and ER), 4 weeks; (B) Embryo formed on callus, 5 weeks;  (C, D) Embryos formation, 7 weeks; (E, F) Embryo germination on selection medium, 9-10 weeks; (G) Transgenic alfalfa plants transferred to Magenta box, 12 weeks; (H) Transgenic alfalfa plant transferred to soil, 18 weeks; (I) Potted transgenic alfalfa plants in a growth chamber, 28 weeks.


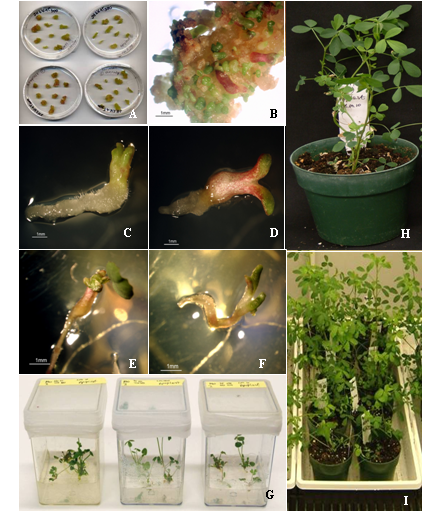

Supplement: Additional file 11 — Schematic map of the 5,103 bp pEACH vector based on pPZP100[36]. Modifications described in the Methods section. Par A, par A MYB recognition sequences. [file 1754-6834-7-39-S11.docx]
